# Sleeping for One Week on a Temperature-Controlled Mattress Cover Improves Sleep and Cardiovascular Recovery

**DOI:** 10.3390/bioengineering11040352

**Published:** 2024-04-03

**Authors:** Nicole E. Moyen, Tatiana R. Ediger, Kathryn M. Taylor, Erin G. Hancock, Lucas D. Holden, Emma E. Tracy, Philip H. Kay, Charles R. Irick, Kevin J. Kotzen, David D. He

**Affiliations:** 1 Eight Sleep, Inc., New York, NY 10010, USA; 2 K.T. Research Consulting, Boston, MA 02215, USA

**Keywords:** polysomnography, sex differences, sleep quality, sleep satisfaction, temperature regulation, thermal comfort zone, thermoregulation

## Abstract

Body temperature should be tightly regulated for optimal sleep. However, various extrinsic and intrinsic factors can alter body temperature during sleep. In a free-living study, we examined how sleep and cardiovascular health metrics were affected by sleeping for one week with (Pod ON) vs. without (Pod OFF), an active temperature-controlled mattress cover (the Eight Sleep Pod). A total of 54 subjects wore a home sleep test device (HST) for eight nights: four nights each with Pod ON and OFF (>300 total HST nights). Nightly sleeping heart rate (HR) and heart rate variability (HRV) were collected. Compared to Pod OFF, men and women sleeping at cooler temperatures in the first half of the night significantly improved deep (+14 min; +22% mean change; *p* = 0.003) and REM (+9 min; +25% mean change; *p* = 0.033) sleep, respectively. Men sleeping at warm temperatures in the second half of the night significantly improved light sleep (+23 min; +19% mean change; *p* = 0.023). Overall, sleeping HR (−2% mean change) and HRV (+7% mean change) significantly improved with Pod ON (*p* < 0.01). To our knowledge, this is the first study to show a continuously temperature-regulated bed surface can (1) significantly modify time spent in specific sleep stages in certain parts of the night, and (2) enhance cardiovascular recovery during sleep.

## 1. Introduction

Good-quality sleep is important for memory consolidation [1], learning [2], metabolic regulation, immunity, and restoration [3], while poor-quality sleep is associated with various problems such as dementia [4], metabolic dysregulation [5], and cardiovascular disease [6]. One-third of Americans get less than seven hours of sleep per night, and over 70 million Americans have a sleep disorder [7]. Body temperature is one of the main factors that affects sleep quality and, therefore, must be tightly regulated for optimal sleep [8,9,10].

Maintaining skin temperatures within 33.5–35.5 °C during sleep is crucial, as deviations outside of this range increase sleep disturbances, decrease deep sleep, and reduce total sleep time (TST) [11,12,13,14,15,16]. Thermal comfort throughout the night also positively correlates with perceived sleep quality [17,18,19]. Previous studies have manipulated ambient temperature based on sleep stages to enhance thermal comfort and perceived sleep quality [17,18,19,20]. However, maintaining an optimal skin temperature range during sleep can be difficult if hot or cold ambient bedroom temperatures cannot be offset by clothing, bedding, and/or HVAC systems [11,13,21]. Moreover, aging and specific conditions like major depression and narcolepsy can alter thermoregulatory abilities, making it difficult to obtain good-quality sleep [16,22,23]. Previous studies have employed a variety of methods to modify body temperatures to promote sleep onset and/or improve sleep quality, including manipulating ambient temperature [24,25], warming the extremities [26], modifying clothing [21] or bedding layers [23], using electric blankets [27], using a thermal suit during sleep [28], cooling the head [29], or sleeping on a high heat capacity mattress [30,31,32].

The ideal thermoregulatory solution during sleep should be cost-effective and practical for daily use. Altering the bed surface temperature is one such solution. One implementation is a high-heat capacity mattress (HHCM), which passively absorbs body heat during the early part of the night. HHCM studies show increased deep sleep during the first sleep cycle and a lower resting heart rate (HR), which is attributed to slightly lower skin and core temperatures early in the night [30,31,32,33]. However, there were no added benefits from HHCM after the first sleep cycle, likely because HHCM and normal mattresses reach similar temperatures during the second half of the night. HHCM also cannot individualize temperatures based on unique physiology; it is a “one-size-fits-all” solution [30,31,32,33]. For example, men and women find similar skin temperatures to be comfortable during sleep [34,35]; however, women have generally lower skin temperatures compared to men due to their lower metabolic rate [36]. Thus, women prefer ~1.5–2 °C warmer microclimates and ambient temperatures during sleep compared to men and are more sensitive to deviations outside of the thermal comfort zone [37,38]. Therefore, an improved solution would regulate temperature for each individual throughout the entire sleep duration, which would promote customized and consistent sleep improvements.

Compared to HHCM, the Eight Sleep Pod is a temperature-regulated mattress cover that provides individualized temperature regulation (i.e., selection of temperatures ranging from 13 to 43 °C) on each side of the bed at three different points in the night, allowing for continuous temperature regulation throughout the night. Therefore, the goal of this study was to investigate whether the Eight Sleep Pod would improve sleep and cardiovascular metrics. As previous research on HHCM has found lower resting HR during sleep [30,31,32], we hypothesized that sleeping on the Pod at cooler temperatures would lead to lower HR and potentially higher HRV. Moreover, we hypothesized that the results would be dependent on biological sex, as women need warmer temperatures during sleep compared to men [37,38] and have different sleep architectures [39]. Additionally, previous studies have only evaluated these temperature interventions over a single night, which precludes the evaluation of any carry-over or long-term effects of sleeping on a temperature-regulated bed. This study accounts for such effects by alternating the bed temperatures off and on across a two-week period and recruiting an equal number of men and women to participate in the study. To our knowledge, this is the first study to show that a continuously temperature-regulated bed can (1) modify sleep stages at specific points in the night, (2) improve thermal comfort and perceived sleep quality, and (3) enhance cardiovascular recovery during sleep.

## 2. Methods

**Figure 1:**
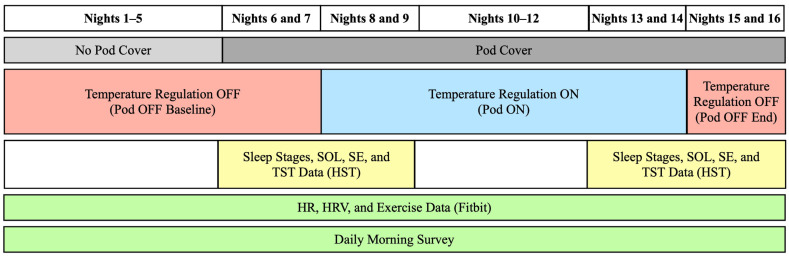
Experimental Design Schematic. Subjects (*n* = 54) spent the first seven nights with temperature regulation off (Pod OFF Baseline) and then spent seven nights with temperature regulation on (Pod ON), followed by the last two nights with temperature regulation off (Pod OFF End). “No Pod Cover” means that subjects slept on their own bed without the Pod cover installed. “Pod Cover” means that subjects were sleeping with the Pod cover on their bed, either with the temperature regulation on or off. Sleep stages, sleep onset latency (SOL), sleep efficiency (SE), and total sleep time (TST) were recorded on Nights 6–9 and 13–16 through a home sleep test device (HST). Throughout the entire study, sleeping HR and HRV, along with daily exercise, were recorded via a Fitbit. Ratings of perceptual comfort and sleep quality were recorded via a daily morning survey.

The main goal of this study was to understand how sleeping on an active temperature-controlled mattress cover (the Eight Sleep Pod; see Supplementary Materials Figure S1) affected sleep, cardiovascular, and perceptual outcomes in free-living conditions. To answer this question, subjects were instructed to sleep on the Pod (in their homes) with the temperature regulation turned off (Pod OFF) for one week in order to collect baseline physiological and perceptual responses. Next, subjects slept with the Pod’s temperature regulation (Pod ON) for one week to assess the impact of continuous temperature regulation during sleep on cardiovascular, sleep, and perceptual metrics. Cardiovascular metrics included HR and heart rate variability (HRV) from Fitbit devices during sleep (see details below). Sleep metrics included time spent in each sleep stage, TST, sleep onset latency (SOL), and sleep efficiency (SE) from a home sleep test device (see details below). Perceptual metrics included thermal comfort and sensation, sleep satisfaction, calmness, refreshedness, ease of falling asleep and waking up, and sleep quality. Lastly, subjects slept for two nights with Pod OFF at the end of the two-week period to determine whether any physiological changes persisted after the one-week intervention with Pod ON. See Figure 1 for a schematic of the experimental design. To maximize ecological validity, all subjects completed the study in their normal bedrooms under free-living conditions (e.g., no experimental control over ambient temperature, lighting, or sleep/wake times). However, subjects were asked to maintain similar bedroom conditions and habits throughout the study.

### 2.1. Subject Characteristics

A total of 75 subjects were recruited for this study, and 69 subjects completed the study. After filtering out nights with noisy or missing EEG data and non-temperature-compliant subjects (see below for filtering criteria), 54 subjects successfully completed the 16-night study (see Results for anthropometric details). Subjects were excluded if they were unavailable to sleep on the Pod for two consecutive weeks, were under 18 years old, or had an unsupported bed size. Subjects were also excluded if they reported having any of the following criteria: a pacemaker, restless leg syndrome, an apnea–hypopnea index (AHI) above 30, insomnia, or taking beta-blockers. Additionally, subjects were excluded if they reported that they normally sleep less than four hours per night on more than three days per week. Seven subjects reported conditions (heart and respiratory) not listed in the exclusion criteria: six subjects reported a respiratory condition, and one subject reported both heart and respiratory conditions (Table 1). Subjects’ self-reported ethnicities were African American (1), Latino (2), Caucasian (45), multiple ethnicities (5), and other (1). All subjects provided written consent to participate in the study, approved by the Sterling Institutional Review Board (IRB identification number 10282).

### 2.2. Experimental Design

Before starting the study, subjects filled out a medical history survey (see Supplementary Materials) and the Pittsburgh Sleep Quality Index (PSQI, [40]; pre-PSQI). The pre-PSQI asks subjects about their normal sleeping habits over the previous month (see Supplementary Materials). The PSQI generates a global PSQI score and seven sub-component scores for subjective sleep quality, sleep onset latency, sleep duration, sleep efficiency, sleep disturbances, use of sleeping medications, and daytime dysfunction.

#### 2.2.1. Pod OFF Baseline (Week 1)

For the first seven nights during Pod OFF, baseline physiological and perceptual data were collected. To collect sleeping HR and HRV along with exercise data, subjects were mailed a Fitbit Versa 2 (Fitbit Inc., San Francisco, CA, USA) to start wearing on Night 1. Subjects wore their Fitbit throughout the entire study and were asked to log any exercise sessions that they completed. Subjects were also sent a daily survey each morning, which included perceptual questions about thermal comfort, sleep satisfaction, and ease of falling asleep and waking up (see Supplementary Materials).

Subjects slept on their own mattresses for Nights 1–5 until the Pod was installed by an Eight Sleep research associate on Night 6. For Nights 6 and 7, subjects slept on the Pod with the temperature OFF. Sleep stages, SOL, SE, TST, and deep and REM sleep onset latencies were collected on these nights via a home sleep test device (HST; Zmachine Synergy, General Sleep Corp., Cleveland, OH, USA).

#### 2.2.2. Pod ON (Week 2)

For the next seven nights (Nights 8–14), subjects slept on the Pod with the temperature regulation on (Pod ON). See below for details about how the Pod’s temperature regulation works. To assess whether there were any short-term vs. longer-term changes in sleep with Pod ON, subjects wore the HST on Nights 8–9 and 13–14, which corresponded with the first two nights and last two nights of the one-week intervention of Pod ON. Subjects continued to wear the Fitbit and fill out the daily surveys for Nights 8–14. After Night 14, subjects filled out a post-Pod PSQI (see Supplementary Materials) that asked subjects questions about their sleep habits during the week of Pod ON. The post-Pod PSQI had slightly altered questions to account for the adjusted timeline (one week vs. one month). Therefore, only a subset of PSQI subcomponents could be compared from pre- to post-PSQI (see Section 2.5 for more details).

#### 2.2.3. Pod OFF End (Final Two Nights)

On Nights 15 and 16, subjects slept with Pod OFF again to determine whether there were any carry-over effects of sleeping with Pod ON. Subjects wore the HST for the last two nights, filled out the daily surveys, and continued to wear Fitbit for resting HR and HRV measurements. On day 17, study equipment was collected from the subjects.

#### 2.2.4. Temperature Compliance

To ensure subjects complied with the experimental design for Pod ON and Pod OFF, their Pod temperature data were monitored daily. The temperature data were recorded in real-time and stored in a relational database (Postgres). These data were queried for each subject over the course of the study to determine their temperature values for each night. A subject was temperature-compliant if they slept with Pod OFF for their first two nights on the Pod (Nights 6 and 7), slept with Pod ON for the subsequent seven nights (Nights 8–14), and then turned the Pod OFF for the final two nights (Nights 15 and 16). Subjects were asked to repeat a night if they did not follow this schedule. If a subject had to repeat a night for any reason, they could still be considered temperature-compliant if they had more than two nights at the beginning with Pod OFF, at least six nights during Week 2 with Pod ON, and two or more nights with Pod OFF at the end. Only seven subjects had to repeat a night during the study to remain temperature-compliant.

### 2.3. Physiological Data

#### 2.3.1. The Eight Sleep Pod

The Eight Sleep Pod has two main capabilities: (1) continuously regulate water temperature flowing through the mattress cover during the night, and (2) collect biometric data (HR, HRV, respiratory rate, and sleep staging data). These functionalities occur independently on both sides of the bed while the person sleeps. The Pod consists of a hub that sits beside the bed and a cover that fits over the mattress like a thick fitted sheet. Inside the cover, water flows through a water mat to heat and cool the bed according to the subject’s preference. The Pod temperature is controlled through the Eight Sleep application (iOS and Android) using a temperature dial. The water temperature circulating throughout the Pod was programmed by each subject through the app and can achieve water temperatures ranging from ~13 to ~43 °C, which corresponds to a range of −10 to +10 on the temperature dial in the Eight Sleep app. Each person sleeping on the Pod can independently program the temperature on their half of the bed. A temperature profile is customizable and consists of three temperature settings that automatically cycle throughout the night. The first temperature setting, the Bedtime Phase, lasts from the time the person gets into bed until 15 min after persistent sleep is detected. The second temperature setting, the Early Phase, lasts for four hours after the Bedtime Phase ends. The third temperature setting, the Late Phase, lasts from the end of the Early Phase until waking.

To help subjects quickly select temperature settings that were neutral and minimize any adjustment period of sleeping on the Pod, the research associates recommended a different temperature profile for women vs. men. Women were recommended −1, 0, and +1, while men were recommended −2, −1, and 0, which correspond to water temperatures of approximately 26, 27, and 29 °C vs. 25, 26, and 27 °C, respectively. Although subjects were given these recommendations, they were allowed to create any temperature profile they preferred during Pod ON and then continue to adjust their profile throughout the week as desired. We purposefully did not control Pod temperatures to maximize ecological validity and mimic how people might use the product in the real world. Means ± SD of subjects’ temperature settings with Pod ON can be found in Supplementary Materials Table S1.

Note that although physiological data from the Pod were recorded throughout sleep, none of these data were used in this manuscript. This was to ensure that any findings during Pod ON were based on independent third-party devices (Fitbit and HST).

#### 2.3.2. HST

For eight nights, subjects wore the Zmachine Synergy, an HST that records the following: single-channel electroencephalogram (EEG) from three electrodes, respiratory effort via respiratory inductance plethysmography, respiratory airflow via nasal cannula, oxygen saturation via pulse oximeter, heart rate via photoplethysmography, and body position via tri-axis accelerometer (rotation and tilt in degrees). The Zmachine sleep monitoring system takes the EEG signal and generates sleep stages via an automated single-channel EEG sleep staging algorithm, Z-ALG, that is FDA-cleared, with a Cohen’s kappa agreement of 0.72 [41]. At the Pod installation (for subjects without a Pod) or via zoom (for subjects with a Pod), an Eight Sleep research associate guided subjects on proper HST setup. To ensure maximum adherence to the EEG electrodes throughout the night, subjects were instructed to attach the three EEG electrodes to the skin (behind the ears and back of the neck) for ~20 min before attaching the wires. Subjects fit the HST respiratory belt over light clothing on their breastbone/nipple line and wore the nasal cannula taped to their cheeks and secured behind their ears. Subjects were additionally provided with General Sleep videos and manuals on proper HST installation, as well as Zoom call check-ins and warnings on actions that may impede data collection. If subjects reported any issues with the HST in the daily survey, the research associate would follow up with them to ask questions and help mitigate any repeated issues.

All data from the HST were exported as an EDF file and pre-processed in the EDF browser (version 2.0) [42]. The sleep stages are stored as annotations in the EDF file in 30 s epochs. The stages are wake (W), combined light sleep (N1 and N2), combined deep sleep (N3 and N4), Rapid Eye Movement (REM; R), and inconclusive (“?”). If there were fewer than three hours of sleep stage data, we classified the night as invalid. If a subject’s night was invalid due to HST checks or temperature compliance checks (see above), then they would be asked to repeat a night. Out of our total population, nine subjects repeated a night with the HST during the study; however, this repeat night was only included if it was in proper sequence with when the Pod was ON or OFF. Furthermore, within Pod ON, the repeat HST night had to occur at the beginning or end of the week because there were three days without HST in the middle of the week.

#### 2.3.3. Fitbit HR, HRV, and Exercise Data

Since the HST was only worn on 8 out of the 16 nights and the Pod was only used on 11 out of the 16 nights, we used Fitbit Versa 2 to evaluate changes in HR and HRV across the course of the study since it was worn continuously for all 16 nights. From the Fitbit data, we analyzed sleeping HR and HRV, along with physical activity data, including time spent in HR zones and total daily steps. The Fitbit Versa has been validated for HR compared to electrocardiogram (ECG) *r* = 0.91 [43], and Fitbit HRV is statistically similar to Polar H10 ECG (paired *t*-test: *p* = 0.28) [44]. See Supplementary Materials for details on the Fitbit exercise zones. After completion of the study, the Eight Sleep research team accessed the Fitbit data through a combination of the free Fitbit data web export (via logging into each subject’s account that the research team created for them) and through Terra API (England, UK) integration in the Eight Sleep app. Through these two data sources, we obtained daily information for each subject, including minutes spent in each HR zone, total steps, minimum sleeping HR, and median sleeping HRV. Fitbit sleep data were not used for any analyses except to serve as a secondary check for HST data when there were substantial missing data due to noisy EEG (see details below).

### 2.4. Data Quality Checks and Data Processing

#### 2.4.1. HST Post-Processing

Further processing was conducted with the HST data in order to calculate SOL, LPS (latency to persistent sleep), TST, REM sleep latency, deep sleep latency (Ldeep), and SE for the entire night. In order to calculate SOL, we first found sleep onset. Sleep onset was defined as the first occurrence of sleep in any sleep stage within the first hour with at least 10 min of sleep in any sleep stage. Since subjects turned on the HST right before going to sleep, SOL was found using the difference in minutes between sleep onset and the start of the nightly HST recording. SOL itself is often calculated using the first epoch of stage 2 sleep [45], but Z-ALG returns stages 1 and 2 together as light sleep [41], so we cannot distinguish between stage 1 and stage 2. To overcome this, we found the first hour where the individual was asleep for more than 10 min. Within this first hour of substantial sleep, we found the exact time when sleep started. The 10 min threshold avoids counting intermittent light sleep as the start of the sleep period. This method was manually reviewed by two individuals blinded to Pod OFF vs. Pod ON. LPS was defined as the start of the HST recording until the first occurrence of 10 consecutive minutes of sleep in any Phase [46]. If the HST was removed before stopping the recording, there could be upwards of 10 h of “?” stages, so the end of the file (sleep offset) was defined as the last occurrence of REM, deep, light, or wake–sleep stages. TST was found by taking the sum of all minutes spent in REM, deep, and light sleep from sleep onset until the end of the file. The number of minutes spent in REM, deep, light, wake, and “?” stages was calculated by taking the total number of 30 s epochs in each stage and dividing that by two. REM latency was defined as the amount of time between SOL and the first occurrence of REM sleep. The number of awakenings was defined as the number of awake periods following SOL, which was calculated by finding the distinct occurrences of consecutive wake stages [47]. SE was defined as TST divided by the duration of the HST recording up until the calculated end of the file, excluding from both the numerator and denominator the minutes spent in the “?” stage [48].

Additionally, we calculated TST, the total minutes spent in each sleep stage, the percentage of time spent in each sleep stage relative to TST, and the total number of awakenings for both the Early Phase and Late Phase that correspond with temperature changes on the Eight Sleep Pod (defined above). To calculate these metrics for each Phase, the 30 s epochs were divided into their respective Phases. From there, the same method as above was used to calculate the number of minutes spent in REM, deep, light, and wake stages, respectively, for each Phase. 

#### 2.4.2. Filtering Methods

For both the HST and Fitbit datasets, each night was matched with the subject’s temperature status (i.e., Pod ON or Pod OFF) for that night. The temperature status categories are: (1) Pod OFF Baseline (first seven nights of baseline with Pod OFF or not yet installed); (2) Pod ON beginning (first four days of the week with Pod ON); (3) Pod ON end (last three days of the week with Pod ON); and (4) Pod OFF End (final two days of the experiment with Pod OFF). After applying extensive filtering methods for Fitbit and the HST (see Supplementary Materials for more details), 44 out of 56 subjects were included in the HST analysis and 54 out of 56 subjects for the Fitbit analyses (see Table 1).

### 2.5. Statistical Analysis

To determine the necessary sample size for this study, we evaluated a dataset comprising 300+ people who wore an Oura ring for at least one week before and after purchasing an Eight Sleep Pod (i.e., going from Pod OFF to Pod ON). In analyzing these data, we found that resting HR was lower, on average, by 0.9 bpm. We used the mean and SD from this dataset from Pod OFF to Pod ON to do a power calculation (power of 0.8, alpha = 0.05, two-tailed paired *t*-test, effect size = 0.41), which led to a minimum sample size of 49 subjects needed to detect a difference of 0.9 beats/min in HR from Pod OFF to Pod ON.

To obtain each subject’s temperature data for each Phase (Bedtime, Early, and Late) for Pod ON, a weighted mean temperature was calculated. This is because if a subject changed their temperature in the middle of a Phase, a weighted mean better represented the overall temperature they experienced by accounting for the time spent at each temperature during that Phase. For Pod OFF nights, there were no temperatures, but the total time spent in each sleep stage was still binned into Early and Late Phases by using the same time cutoffs for each Phase as when Pod ON (defined above). Cool vs. warm Pod temperatures were defined by dichotomizing Pod temperatures for men and women separately at the median for each of the three temperature Phases on the Pod (Bedtime, Early, and Late Phases). For each Phase, temperatures below the median were defined as cool temperatures, and those above the median were defined as warm temperatures. The median Pod temperatures during Pod ON for women vs. men at Bedtime were 25.8 °C vs. 23.4 °C, respectively; for the Early Phase, were 26.0 °C vs. 25.4 °C, respectively; and for the Late Phase, were 28.6 °C vs. 26.8 °C, respectively.

Linear mixed models were used to evaluate changes in the cardiovascular recovery data (HR and HRV) and time spent in each sleep stage for Pod OFF vs. ON. Pod temperature was analyzed in three separate models: (1) a binary variable (ON vs. OFF), (2) a categorical variable where Pod OFF Baseline was compared to sleeping with Pod ON or Pod OFF End, and (3) a second categorical variable where all Pod OFF days were compared to sleeping at cool vs. warm Pod temperatures when Pod ON. Models evaluating cool vs. warm temperatures were run separately in men and women due to the reported temperature differences between sexes during sleep [37,38]. The linear mixed model analysis meant that for each night, the individual was categorized as a cool or warm sleeper, and this categorization changed based on how they set their temperature each night. Sleep-stage specific analyses were restricted to the subset of subjects who had at least one night of HST data in Pod OFF Baseline and Pod OFF End and at least two nights of HST data during Pod ON (*n* = 44; see detailed explanation above).

Cumulative link mixed models (CLMM) were used to estimate odds ratios (OR) and 95% confidence intervals (CI) for the relationship between Pod temperature as well as the ordinal daily perceptual questions and the six PSQI components [40]. The PSQI analysis evaluated changes in six out of the seven PSQI sleep components: sleep quality, sleep onset latency, sleep duration, sleep efficiency, sleep medication use, and daytime dysfunction. Changes in the sleep disturbances component and global PSQI score were not evaluated because the validated scoring for these metrics uses responses that require an assessment over one month, which was not possible to assess after one week of Pod ON. Pod temperature was analyzed in separate models as (1) a binary variable (Pod ON vs. OFF) and (2) a categorical variable where Pod OFF Baseline was compared to sleeping at a cool vs. warm Pod temperature. Each model was fitted with a random intercept to account for individual correlations. For each of the sleep components in the PSQI, an increased score is considered a worse sleep outcome. Therefore, an OR < 1 for any PSQI component indicated a sleep improvement compared to the Pod OFF Baseline. Conversely, a higher score on the daily perceptual questions, except for the thermal sensation question, indicated better sleep outcomes. In this case, an OR >1 indicated improvements in sleep compared to the Pod OFF Baseline. With respect to the daily survey question on thermal sensation, an OR < 1 indicated that, on average, subjects felt warmer during sleep, whereas an OR > 1 indicated subjects felt cooler during sleep compared to Pod OFF.

In the subset of individuals with HST data (*n* = 44), CLMM were used to evaluate the relationship between the daily perceptual questions and changes in HST-measured sleep metrics to evaluate the mind-body connection. Changes in sleep metrics were calculated for each individual within each temperature Phase by subtracting the average time spent in the sleep stage during Pod OFF from the time spent in each night’s sleep stage with Pod ON. These values were dichotomized, where a change score > 0 indicated an improvement in sleep with Pod ON, and a change score < 0 indicated a reduction in sleep with Pod ON. For the change in wake time and number of awakenings, values were inverted so that negative change scores were considered an improvement in sleep. The goal of these analyses was to demonstrate whether improvements in the sleep metrics (measured via HST) actually resulted in perceived sleep improvements.

For all models, alpha was set at 0.05. All models were evaluated for effect modification by sex using a multiplicative interaction term. All results were evaluated and visualized using R statistical software (version 4.2.2) [49], and all pre-processing of the data was performed with Python (version 3.7).

## 3. Results

### 3.1. Subject Characteristics and HST and Fitbit Frequencies

A total of 54 subjects completed the study (27 females and 27 males, mean ± SD age  = 36.0 ± 14.4 y; range 21–74 y) [Table 1]. Subjects completed 160 nights of HST and 466 nights of Fitbit with Pod OFF, and 158 nights of HST and 367 nights of Fitbit with Pod ON. A total of 62.5% of subjects reported having a consistent bed partner. The population was predominantly healthy, with only one subject (2%) and seven subjects (13.0%) reporting heart conditions and respiratory conditions, respectively. Approximately nine subjects (17%) reported using sleep medication at baseline; however, the study population comprised normal (healthy) sleepers according to a similar global PSQI score to the normal population [50] [Table 1]. Female subjects, on average, had a significantly higher global PSQI compared to male subjects by 0.2 points (*p* = 0.002); however, both sexes were still considered healthy sleepers.

### 3.2. Effect of Pod Use on HR and HRV

**Figure 2:**
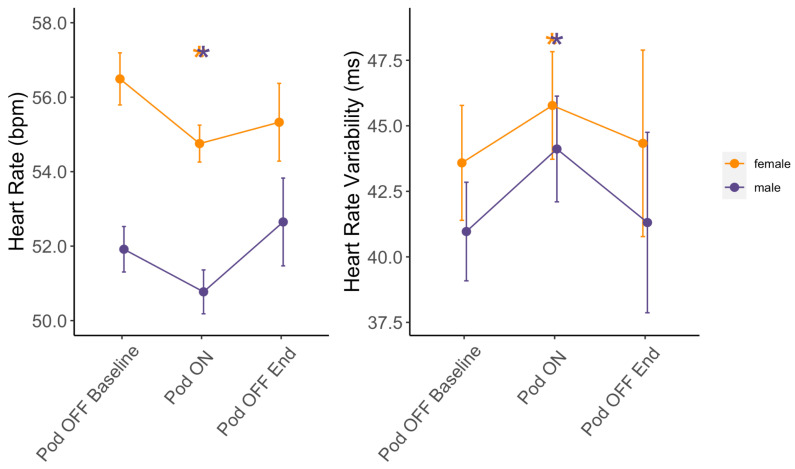
Changes in HR and HRV based on Pod temperature status. Means ± SE for minimum sleeping HR and median sleeping HRV at Pod OFF Baseline, Pod ON, and Pod OFF End stratified by sex (females in orange and males in purple). * Indicates a significant difference in linear mixed models from Pod OFF Baseline at *p* < 0.05.

Minimum sleeping HR was significantly lower on average by 1.2 bpm (*p* < 0.001) and median sleeping HRV was significantly higher on average by 2.0 ms (*p* = 0.009) when sleeping with Pod ON vs. Pod OFF Baseline [Figure 2 and Supplementary Materials Table S2]. The median and mean percent changes in HR from Pod OFF to ON were −2.4% and −1.9%, respectively, with a maximum percent change of −13.2%.

The median and mean percent changes in HRV from Pod OFF to ON were 5.2% and 7.1%, respectively, with a maximum percent change of 16.5%.

HR and HRV were similar between Pod OFF Baseline vs. Pod OFF End (all *p* > 0.05), indicating changes with Pod ON were acute responses. There were no significant differences between men and women with regard to changes in minimum HR and median HRV with Pod ON (all *p* > 0.05). Notably, these changes in HR and HRV were not a result of changes in exercise habits from Pod OFF Baseline to Pod ON, as there were no significant differences in the time subjects spent exercising in the Fat Burn Zone, Cardio Zone, or number of steps taken each day (all *p* > 0.05) [Supplementary Materials Table S3].

For men sleeping at cooler temperatures during the Early Phase, minimum HR was significantly lower by 1.23 bpm (*p* = 0.020), and median HRV was significantly higher by 2.87 ms (*p* = 0.039) [Supplementary Materials Table S4]. Similar effects were not observed when men slept at warmer temperatures during the Early Phase (all *p* > 0.05). During the Late Phase, men’s minimum HR at both warm and cool temperatures was significantly lower on average by 1.06 bpm (*p* = 0.046) and 1.02 bpm (*p* = 0.045), respectively [Supplementary Materials Table S4]. However, the temperature had no effect on HRV during the Late phase. Changes in HR and HRV for women were unaffected by sleeping temperature for the Early and Late Phases (all *p* > 0.05) [Supplementary Materials Table S4].

### 3.3. Effect of Pod Use on Sleep Metrics

#### 3.3.1. Men

**Figure 3:**
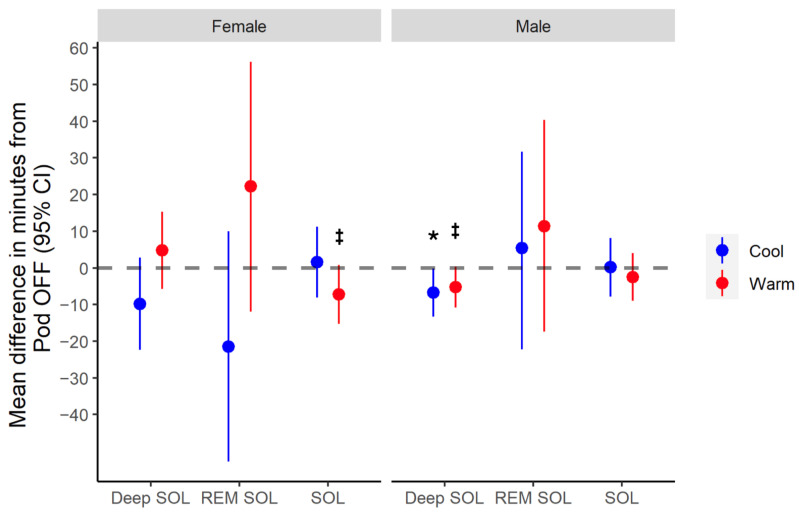
Changes in sleep latency metrics from Pod OFF to ON for cool vs. warm Bedtime temperatures. Mean difference and 95% confidence intervals for the change in sleep onset latency (SOL) and Deep SOL metrics from Pod OFF Baseline vs. Pod ON sleeping at cool or warm Bedtime temperatures, stratified by sex. REM SOL represents the mean difference from Pod OFF to cool vs. warm Early-Phase temperatures. * Indicates a significant difference from Pod OFF Baseline at *p* < 0.05. ^‡^ Indicates trend towards being significantly different from Pod OFF Baseline at *p* < 0.10.

Sleeping at cool Bedtime temperatures led to a significantly faster deep sleep onset latency vs. Pod OFF (mean change = −6.70 min, *p* = 0.045), whereas sleeping at warm Bedtime temperatures did not (*p* = 0.066) [Figure 3 and Table 2].

**Figure 4:**
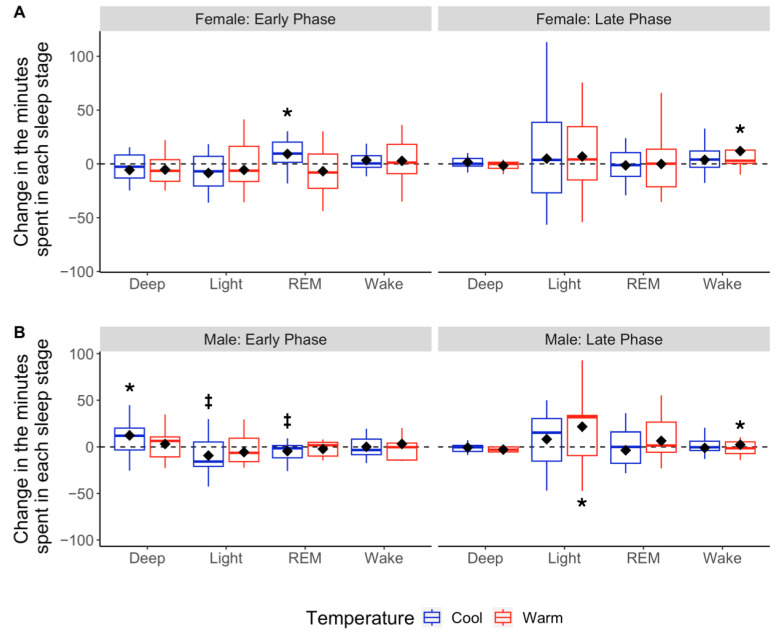
Changes in absolute sleep stage duration based on cool vs. warm sleepers in Early and Late Phases. Boxplots depicting distributions of changes in time (min) spent in each sleep stage for Pod OFF vs. cool (blue) or warm (red) temperatures during the Early and Late Phases for females (**A**) and males (**B**). The black diamonds denote sample means, and the horizontal lines within the boxes denote medians. The dotted line at 0 indicates no change from Pod OFF Baseline to Pod ON. * Indicates a significant difference in linear mixed models from Pod OFF Baseline at *p* < 0.05, and ^‡^ indicates trending toward significance at *p* < 0.10.

**Figure 5:**
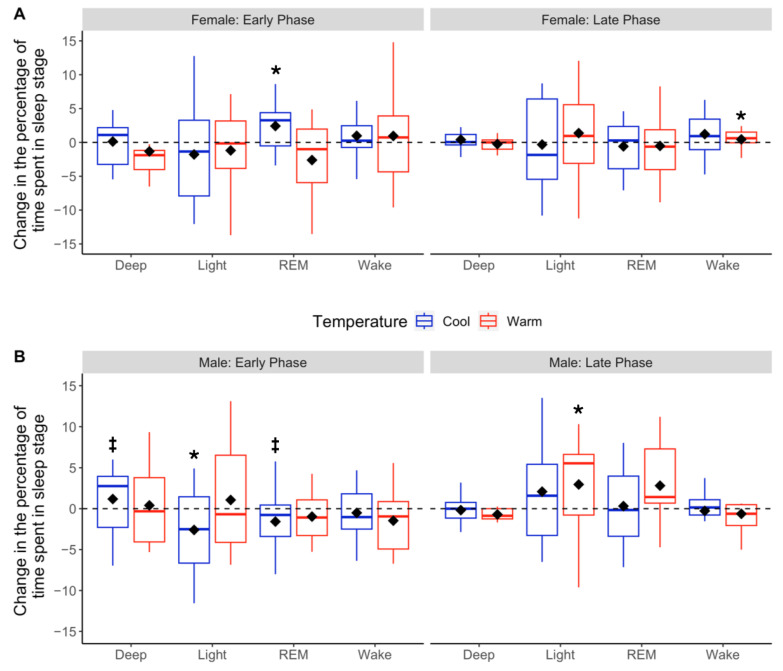
Changes in the percentage of total sleep time spent in each sleep stage based on cool vs. warm temperatures in Early and Late Phases. Boxplots depicting distributions of changes in the percentage of time spent in each sleep stage for Pod OFF vs. cool (blue) or warm (red) temperatures. Early Phase (left-hand panels) and Late Phase (right-hand panels) are separated for females (**A**) and males (**B**). The black diamonds denote means, and the horizontal lines within the boxes denote medians. The dotted line at 0 indicates no change from Pod OFF Baseline to Pod ON. * Indicates a significant difference in linear mixed models from Pod OFF Baseline at *p* < 0.05, and ^‡^ indicates trending toward significance at *p* < 0.10.

Sleeping at cool Early-Phase temperatures increased deep sleep by 14.3 min on average (*p* = 0.003), which equated to a 22% increase in Early Phase deep sleep compared to Pod OFF [Figure 4A and Table 2]. This change trended towards significance (*p* = 0.051) with a 2.78% increase in the percentage of total sleep time spent in deep sleep [Figure 5A and Table 2]. This increased time spent in deep sleep for men with Pod ON was significantly higher than that of women (*p* = 0.005). Additionally, the increase in men’s deep sleep at cooler temperatures came as a tradeoff for non-significant, but likely physiologically meaningful, changes in REM (−6.7 min or −18% on average) and light sleep (−8.39 min or −7% on average; both *p* = 0.07). Sleeping at warm Early-Phase temperatures did not change sleep architecture compared to Pod OFF (all *p* > 0.05) [Table 2]. Men who had lower baseline deep sleep (Pod OFF) had larger deep sleep improvements with Pod ON (*r*^2^ = 0.40, *p* = 0.018). The relationship is inverse so that for every 1 min less of baseline deep sleep, there was a 0.4% increase in the total sleep time spent in deep sleep with Pod ON.

Sleeping at warm Late-Phase temperatures increased light sleep by 23.4 min on average (~19% increase; *p* = 0.023) but also slightly increased the number of awakenings (+3 awakenings, *p* = 0.025) compared to Pod OFF [Table 2]. Sleeping at cool Late-Phase temperatures did not alter sleep architecture relative to Pod OFF (all *p* > 0.05) [Table 2].

Overall, TST, SE, SOL, and REM onset latency were unchanged from Pod OFF to ON (TST mean change = +10.0 min, 95% CI = [−0.14, 0.48], *p* = 0.284; SE mean change = −0.01, 95% CI = [−0.009, 0.03], *p* = 0.318; SOL mean change = −1.5 min, 95% CI = [−7.03, 4.04], *p* = 0.599; REM onset latency mean change = 6.5 min, 95% CI = [−14.24, 26.81], *p* = 0.544).

#### 3.3.2. Women

Women who slept at warm Bedtime temperatures fell asleep 7 min faster on average compared to Pod OFF (mean change in SOL = −7.28 min; *p* = 0.079). Although this change was not statistically significant, it is likely physiologically significant. Sleeping at cool Early-Phase temperatures increased REM sleep by 9.2 min compared to Pod OFF (25% increase in Early Phase REM sleep; *p* = 0.033), which equated to a 2.48% increase in the percentage of total sleep time spent in REM sleep [Figure 4B and Figure 5B; Table 2]. The time and percent change in REM sleep for women were significantly higher than those of men (time: *p* = 0.005, percent change: *p* = 0.004). This increase in REM sleep appeared to be a tradeoff for small but statistically insignificant decreases in light sleep (mean change = −6.99 min or −6%, *p* = 0.169) and deep sleep (mean change = −4.90 min or −7%, *p* = 0.305) [Table 2]. Women sleeping at warm Late-Phase temperatures significantly increased wake time by 8.15 min (*p* = 0.044), with no difference in the number of awakenings (*p* = 0.123) vs. Pod OFF [Table 2].

Pod temperatures did not affect TST, SE, REM, or deep sleep onset latencies (TST mean change = −0.24 min, 95% CI = [−0.30, 0.29], *p* = 0.981; SE mean change = −0.01, 95% CI = [0.029, 0.011], *p* = 0.359; REM sleep latency mean change = 0.12 min, 95% CI = [−24.39, 24.70], *p* = 0.993; deep sleep latency mean change = −0.03 min, 95% CI = [−8.721, 8.884), *p* = 0.995) [Figure 3 and Table 2].

**Table 2:** Least squared means and standard errors for each sleep stage, stratified by sex and cool vs. warm temperatures for Pod OFF as a reference compared to Pod ON.

Variable	Men					Women					Effect Modification by Sex: Cool	Effect Modification by Sex: Warm
	OFF (Ref)	Cool		Warm		OFF (Ref)	Cool		Warm			
		lsmean ± SE	*p*-Value	lsmean ± SE	*p*-Value	lsmean ± SE	lsmean ± SE	*p*-Value	lsmean ± SE	*p*-Value	*p*-Value	*p*-Value
**Bedtime**												
SOL	28.5 ± 4.1	28.7 ± 5.1	0.965	26.0 ± 4.6	0.452	31.5 ± 3.9	33.0 ± 5.3	0.756	24.2 ± 4.5	0.079	0.756	0.079
Deep SOL	16.8 ± 2.2	10.0 ± 3.2	**0.045**	11.5 ± 2.7	0.066	18.4 ± 4.3	8.5 ± 6.2	0.124	23.1 ± 5.0	0.377	0.654	0.100
**Early Phase**												
Deep (min)	64.3 ± 4.9	78.6 ± 5.7	**0.003**	70.9 ± 6.3	0.205	68.5 ± 6.5	63.6 ± 7.1	0.305	61.8 ± 7.3	0.194	**0.005**	0.071
Deep (%)	17.3 ± 1.5	20.1 ± 1.7	0.051	18.7 ± 1.9	0.389	17.4 ± 1.7	16.8 ± 1.9	0.669	15.9 ± 1.9	0.292	0.089	0.178
REM (min)	37.2 ± 3.34	30.5 ± 4.0	0.072	35.3 ± 4.5	0.628	35.0 ± 4.2	44.3 ± 4.9	**0.033**	35.5 ± 5.1	0.924	**0.005**	0.702
REM (%)	10.0 ± 0.9	7.8 ± 1.1	0.061	9.1 ± 1.3	0.476	9.3 ± 1.1	11.7 ± 1.3	**0.028**	8.8 ± 1.4	0.686	**0.004**	0.823
Light (min)	113.0 ± 5.9	105.0 ± 6.6	0.075	108.0 ± 7.0	0.341	115.0 ± 6.7	108.0 ± 7.4	0.169	109.0 ± 7.6	0.301	0.843	0.923
Light (%)	30.5 ± 1.9	27.0 ± 2.2	**0.047**	28.4 ± 2.4	0.291	29.8 ± 1.9	28.4 ± 2.1	0.326	28.8 ± 2.2	0.527	0.401	0.664
Wake (min)	22.8 ± 2.2	22.0 ± 2.8	0.754	21.7 ± 3.1	0.713	20.6 ± 3.1	23.5 ± 3.6	0.375	24.7 ± 3.8	0.236	0.412	0.236
REM SOL	111.0 ± 45.9	116.6 ± 51.2	0.694	132.6 ± 81.1	0.443	122.4 ± 64.5	101.8 ± 75.2	0.181	144.6 ± 52.4	0.202	0.200	0.634
**Late Phase**												
Deep (min)	7.3 ± 1.4	7.3 ± 1.6	0.993	4.6 ± 2.0	0.181	9.2 ± 2.1	10.4 ± 2.7	0.654	7.1 ± 2.7	0.460	0.700	0.902
Deep (%)	1.8 ± 0.3	1.8 ± 0.4	0.957	1.1 ± 0.5	0.188	2.3 ± 0.5	2.6 ± 0.6	0.622	1.8 ± 0.6	0.499	0.692	0.771
REM (min)	55.6 ± 5.6	48.7 ± 6.5	0.257	66.5 ± 7.9	0.142	45.6 ± 4.3	48.0 ± 5.5	0.678	47.8 ± 5.5	0.706	0.219	0.328
REM (%)	13.4 ± 1.2	11.6 ± 1.4	0.159	15.2 ± 1.7	0.252	11.4 ± 0.9	11.8 ± 1.2	0.792	11.4 ± 1.2	0.990	0.221	0.346
Light (min)	94.4 ± 6.9	105.0 ± 8.2	0.204	117.8 ± 10.2	**0.023**	105.0 ± 7.1	106.0 ± 8.7	0.967	113.0 ± 8.9	0.405	0.378	0.242
Light (%)	23.1 ± 1.3	25.5 ± 1.6	0.166	28.2 ± 2.0	**0.016**	26.1 ± 1.4	26.1 ± 1.7	0.983	27.5 ± 1.8	0.403	0.300	0.190
Wake (min)	21.0 ± 2.4	18.3 ± 2.8	0.216	24.2 ± 3.4	**0.046**	18.7 ± 3.2	24.0 ± 4.0	0.186	26.9 ± 4.0	**0.044**	0.082	0.377

Note: OFF indicates all nights of Pod OFF as a reference for comparing with Pod ON at cool or warm temperatures for Bedtime, Early, and Late Phases for women and men. See Section 2 for the description of cool vs. warm bins. Bolded values indicate significance at *p* < 0.05. lsmeans: least squared means; SE: standard error; SOL: sleep onset latency; Early Phase: first four hours of sleep after sleep onset; Late Phase: end of Early Phase until waking; Cool: those who slept at cool Pod temperatures during Early or Late Phase (see Section 2 for details); warm: those who slept at warm Pod temperatures during Early or Late Phases (see Section 2 for details).

### 3.4. Effect of Pod Use on the PSQI Components

All PSQI components, except sleep efficiency (*p* = 0.915), significantly improved from Pod OFF to ON (all *p* < 0.05) [Table 3]. In the medical history questionnaire, subjects who reported sleep medication use were primarily taking melatonin, which is typically used to aid in sleep onset. As perceived SOL improved, it is likely that melatonin use decreased as a result.

#### 3.4.1. SOL

After sleeping on the Pod, subjects reported a decrease in perceived SOL overall (*p* < 0.001). Men sleeping at warm Bedtime temperatures reported improvements in perceived SOL compared to Pod OFF (*p* < 0.001), while women perceived significantly faster SOL regardless of their Bedtime temperature (cool: *p* = 0.002; warm: *p* < 0.001) [Supplementary Materials Table S5].

#### 3.4.2. Sleep Efficiency

Perceived SE significantly improved for men sleeping at warm Pod temperatures across all phases (Early Phase: *p* = 0.009; Late Phase: *p* = 0.013). There was a non-significant trend for women sleeping at cooler Early-Phase temperatures (*p* = 0.070) and warm Late-Phase temperatures (*p* = 0.084) to perceive better SE [Supplementary Materials Table S5].

#### 3.4.3. Medication Use and Daytime Dysfunction

Medication use significantly decreased from 15 to 9 subjects (11% decrease) with Pod ON (*p* < 0.001) vs. Pod OFF, with no effect modification by sex (*p* = 0.556). Daytime dysfunction was significantly reduced in men regardless of Late-Phase temperatures, and for women regardless of Early or Late-Phase temperatures (all *p* < 0.05) [Supplementary Materials Table S5].

#### 3.4.4. Sleep Quality

Perceived sleep quality significantly improved in men for Pod ON vs. OFF, independent of Early Phase temperature (cool: *p* < 0.001; warm: *p* =.008). However, only men sleeping at cool Late-Phase temperatures reported improved sleep quality compared to Pod OFF (*p* < 0.001) [Supplementary Materials Table S5]. Women reported significantly improved sleep quality with Pod ON regardless of Early- (cool: *p* = 0.001; warm: *p* < 0.001) or Late-Phase (cool: *p* < 0.001; warm: *p* < 0.001) temperatures vs. Pod OFF [Supplementary Materials Table S5].

### 3.5. Effect of Pod Use on Daily Perceptual Questions

#### 3.5.1. Changes in Daily Perceptual Ratings from Pod OFF to ON

During Pod ON, subjects had increased odds of reporting that (1) their sleep was calmer (*p* = 0.039), (2) it was easier to fall asleep (*p* = 0.016), (3) they felt cooler during sleep (*p* < 0.001), and (4) they were more comfortable with their body temperature (*p* < 0.001) [Table 4].

#### 3.5.2. Effect of Pod Temperature on Perceptual Ratings

Women who slept at warmer Bedtime temperatures had 74% increased odds of reporting that it was easier to fall asleep (*p* = 0.001) [Supplementary Materials Table S6]. Men reported significant improvements in their ability to fall asleep with Pod ON, regardless of Bedtime temperature (cool: *p* = 0.004; warm: *p* = 0.006) [Supplementary Materials Table S6].

Women sleeping at warm Early-Phase temperatures had 51% increased odds of improved sleep satisfaction compared to Pod OFF (*p* = 0.010). However, Late-Phase temperatures did not influence women’s sleep satisfaction ratings (cool: *p* = 0.046; warm: *p* = 0.017). Similarly, men sleeping with Pod ON during the Early and Late Phases reported significant improvements in sleep satisfaction, independent of temperature during either Phase (*p* < 0.05 for all comparisons) [Supplementary Materials Table S6].

Women sleeping at warm Early (*p* = 0.018) and Late (*p* = 0.004) Phase temperatures reported feeling significantly more refreshed upon waking, while men did not feel more refreshed with Pod ON vs. OFF (*p* > 0.05 for all comparisons) [Supplementary Materials Table S6].

Women sleeping at cooler Early (*p* < 0.001) and Late (*p* < 0.001) Phase temperatures reported a cooler thermal sensation [Supplementary Materials Table S6]. Thermal comfort was significantly higher for women sleeping at warm Early Phase (*p* = 0.003) and cool Late Phase (*p* < 0.001) temperatures. Whereas for men, thermal comfort significantly improved, and they felt cooler during sleep with Pod ON regardless of their Early or Late Phase temperatures (all *p* > 0.05) [Supplementary Materials Table S6].

#### 3.5.3. Linking Perceptual Ratings with Changes in Sleep Metrics: The Mind-Body Connection

Men with faster SOL (measured via HST) reported it was easier for them to fall asleep (*p* = 0.019) [Table 5 and Supplementary Materials Table S7]. However, women with faster SOL did not perceive falling asleep faster (*p* = 0.301) [Table 5 and Supplementary Materials Table S7]. Improved SE (measured via HST) was associated with feeling (1) calmer about their sleep (*p* = 0.018), (2) more refreshed after waking (*p* = 0.005), and (3) that it was easier to fall asleep (*p* = 0.018) [Table 5]. Subjects with more TST reported that it was easier to wake up in the morning (*p* = 0.040) and that they felt more refreshed upon waking (*p* = 0.049).

Those with more REM sleep, more TST, higher SE, and less wake time reported higher sleep satisfaction (REM: *p* = 0.038; TST: *p* = 0.011; SE: *p* = 0.001; wake time: *p* = 0.026). Subjects reporting higher thermal comfort had approximately two times higher odds of having more light sleep (*p* = 0.023), more TST (*p* = 0.029), and higher SE (*p* = 0.002). Those with higher thermal comfort ratings were also more likely to experience less total wake time (*p* = 0.011) [Table 5].

## 4. Discussion

This is the first study evaluating the effects of continuous body temperature regulation (via a temperature-regulated mattress cover) on sleep stage durations, SOL, TST, SE, and cardiovascular (HR and HRV) outcomes across one week. The notable findings of this study are threefold. First, sleeping at cooler temperatures during the first half of the night increased deep sleep in men and REM sleep in women, whilst sleeping at warmer temperatures in the second half of the night led to more light sleep in men and wake time in women. Second, HR and HRV are improved when sleeping with Pod ON, but this effect is transient, as HR and HRV return to baseline after temperature regulation is removed (Pod OFF). Third, subjects rated their body temperature as significantly more comfortable with Pod ON, resulting in qualitative and quantitative sleep quality improvements.

### 4.1. Impacts of Temperature Regulation during Bedtime Phase

The Bedtime Phase lasts from when the person gets into bed until 15 min after sleep onset. Women who were warm Bedtime sleepers fell asleep 7 minutes faster on average than those sleeping at cooler temperatures [Figure 3]. This finding aligns with research demonstrating that warming the feet, periphery, or neck reduces SOL by promoting heat loss at the periphery, therefore aiding the core temperature drop preceding sleep onset [21,26,51,52,53].

For men, there was no quantitative improvement in SOL [Figure 3], but there was a perceived improvement in SOL [Table 3]. However, sleeping at cooler Bedtime temperatures led to a faster deep sleep onset by approximately 7 minutes [Figure 3]. The lack of physiological change in SOL from Pod OFF to ON in men may be due to individual variability in core and skin temperatures as a result of acclimatization state [54,55], age [56], disease, or race [56], which would modify the Pod temperature needed for optimal SOL. Overall, the 7 min decrease in SOL for women and perceived improvement in SOL for men may be beneficial given that SOL insomnia is prevalent in the general population [54]. Future research should explore the microclimate range that facilitates SOL relative to resting core and skin temperatures.

### 4.2. Impacts of Temperature Regulation during Early and Late Phases

During the Early Phase, men sleeping at cool Pod temperatures averaged 14 more minutes of deep sleep vs. Pod OFF Baseline, while women sleeping at cool Pod temperatures averaged 9 more minutes of REM sleep vs. Pod OFF Baseline [Figure 4]. These improvements in deep and REM sleep came at the cost of reduced time in other sleep stages: increased deep sleep in men was traded for REM and light sleep, while increased REM sleep in women was traded for slight reductions in deep and light sleep [Figure 4 and Figure 5]. To our knowledge, this is the first study to show that modifying skin temperatures during sleep can increase REM sleep. It may be that cooler body temperatures, at least in women, promote REM sleep, as seen in other mammals [57].

In men, the changes in deep and REM sleep with Pod ON promoted a similar amount of time spent in both sleep stages (~19–21% in each stage), which is a characteristic of good sleep quality [58]. Minimal research exists that explores how skin temperature fluctuates with each sleep stage across the night. However, existing research shows that cooling the body during the first half of sleep can increase deep sleep [30,31,32,59], which is similar to what we observed in men sleeping at cooler temperatures but not in women. Further research is needed to explain why sleeping temperatures differentially affect men’s and women’s sleep stage composition.

Cooler temperatures during the second half of the night (Late Phase) did not modify sleep compared to Pod OFF; however, sleeping at warmer temperatures during the Late Phase led to an average increase of 8 minutes of wake time for women and 3 additional awakenings per night for men. It is not surprising that warmer Pod temperatures led to increased wakefulness, as warm environmental sleeping temperatures increase the number of awakenings and wake duration [27,60,61]. Interestingly, we also found that men sleeping at warmer Pod temperatures had 23 more minutes of light sleep vs. Pod OFF [see Figure 4 and Figure 5]. This is a unique finding, given that previous research shows skin temperatures are slightly warmer during REM vs. non-REM sleep stages [62]. As such, it would be hypothesized that warmer Late-Phase temperatures would increase REM, not light, sleep. Therefore, it may be that the optimal body temperature required to promote deep, REM, and light sleep not only differs from each sleep stage during a single sleep cycle [63,64] but also differs throughout the night, in line with the circadian rhythm of core temperature. Research in this area is nascent, and future research should explore the exact skin temperature ranges needed to optimize sleep stages throughout the night.

Similar to previous research [23,28], we found individualized body temperature regulation at different phases of the night can promote time spent in one sleep stage as a tradeoff for another. Additionally, we demonstrated that these findings hold true for men and women across at least one week. Even though the human body can self-regulate sleep stage distribution to a large extent, various types of bedding and clothing, a lack of environmental HVAC [59,65], underlying diseases [66,67], and/or hormonal issues [68,69] can prevent the body from doing so effectively. Therefore, sleeping on a temperature-regulated bed can help keep body temperature within the optimal range required for specific sleep stages, thus restoring time spent in certain sleep stages where there may be a deficit [70]. We recommend men sleep at cool Early Phase and warm Late Phase temperatures to maximize deep and light sleep and women sleep at cool Early and Late Phase temperatures to increase REM sleep and minimize wake time.

### 4.3. Cardiovascular Changes Sleeping on the Pod

Sleeping on the Pod led to improvements in both HR (−2%) and HRV (+7%), indicating improved restoration and recovery. Specifically, men who slept at cool Early-Phase temperatures had larger decreases in HR compared to those who slept at warm Early-Phase temperatures. However, the same temperature-dependent changes were not true for women, as HR was lower regardless of Pod temperatures during the night (Supplementary Materials Table S4). Previous studies in men and women have reported a lower sleeping HR when core and skin temperatures were lowered via an HHCM, presumably by increasing vagal activity and leading to a more restorative state [30,31,32]. Since there was not a clear pattern in our data between the reduction in HR and temperature, it is likely that the Pod temperature required to achieve the lower HR is specific to each individual [54,55,56]. Future work should evaluate the skin temperatures required to lower HR during sleep.

To our knowledge, this is the first study to show an improvement in HRV while sleeping on a temperature-regulated mattress. For men, cool Early-Phase temperatures led to higher HRV, while HRV improvements in Late Phase occurred independent of temperature. Women’s HRV responses were not temperature-dependent. Research shows warmer ambient temperatures lead to lower HRV due to parasympathetic withdrawal [71], while cooler temperatures lower the LF/HF, indicating greater parasympathetic activity and increased HRV [72]. It is likely that sleeping within each individual’s optimal skin temperature range promotes increased parasympathetic activity and therefore higher HRV; however, further research is required to understand the direct impacts of body temperature on HRV during sleep.

Changes in HR and HRV with Pod ON were acute (transient) because HR and HRV went back to baseline (Pod OFF Baseline) during Pod OFF End (Figure 2). This not only demonstrates the potential cardiovascular-recovery benefits of sleeping on a temperature-regulated mattress but also highlights that more research is needed to determine whether sleeping on a temperature-regulated mattress, like the Pod, for more than one week could lead to long-term cardiovascular adaptations such as those seen with exercise training. Generally, cardiovascular adaptations that occur with exercise training or heat acclimation take 2–12 weeks of the repeated stimulus to induce long-lasting changes in HR and HRV [73,74,75]. Since our study intervention was only one week in duration, it may be that sleeping on the Pod and receiving this temperature stimulus nightly for 1–2 months could result in long-term cardiovascular adaptations similar to exercise training or heat acclimation; however, more research is needed.

### 4.4. Women vs. Men Sleeping on the Pod

Women and men both prefer a similar skin temperature range during sleep [35]; however, women need ~1–2 °C warmer ambient temperatures vs. men to achieve the preferred skin temperature range for sleep [37]. On average, women’s Pod temperatures were ~1–2 °C warmer than men’s temperatures across all temperature Phases, which likely helped facilitate women maintaining this tight skin temperature range to optimize their sleep. One of the main differences we found between men and women sleeping on the Pod was that cooler temperatures during the Early Phase led to more REM sleep in women but more deep sleep in men (Figure 4). Previous research has shown that men and women see improvements in deep sleep when skin and core temperatures are lower at the beginning of the night [23,30,31,32]. To our knowledge, we are the first to show increased REM sleep in women sleeping at cooler temperatures in the first four hours of the night. It is unclear why our results differ from previous research. Although not statistically significant, the women in our study had ~2% more deep sleep or ~14 more minutes (~24% of the total night is deep sleep) vs. men. On the contrary, men had 3% more REM sleep, or ~13 more minutes (~20% of the total night is REM sleep) vs. women (Supplementary Materials Table S8). This implies that physiologically, women had more room for improvement in REM sleep while men had more room for improvement in deep sleep. In other words, it could be that men and women were already at their physiological “ceiling” for deep and REM sleep, respectively, and this may explain why cooler temperatures had differing effects on men vs. women. These findings clearly highlight the need for more studies evaluating how temperature differentially affects men’s vs. women’s sleep stages at various points of the night and, furthermore, how baseline sleep architecture modifies these relationships.

Regardless of this discrepancy in sleep stage-related temperature effects, both women and men reported that sleeping on the Pod was better than sleeping on their normal mattresses 64.3% and 75.7% of the time, respectively (Supplementary Materials Figure S2). Thus, an individually temperature-regulated bed cover like the Pod can be beneficial in reducing HR, increasing HRV, and improving quantitative and qualitative sleep quality.

### 4.5. Linking Physiological and Perceptual Data: The Mind-Body Connection

Similar to previous research [76], we found that meaningful physiological changes in sleep did not always correspond with perceived improvements in sleep. For example, men who had significantly improved SOL reported that it was easier for them to fall asleep, whereas the same was not true for women [Supplementary Materials Table S7]. Similar to previous studies [77,78], we found that increased deep sleep did not lead to higher perceived sleep quality or satisfaction. Instead, subjects reported feeling more refreshed upon waking and satisfied with their sleep when getting more TST and REM sleep and less wake time. This is similar to findings from Della Monica et al. [78], who also found that increased REM and decreased wake were related to improved sleep quality ratings. As seen in previous studies [76,79], SE improvements (measured via HST) were the most strongly related variable to perceived improvements in sleep quality [Table 5]. Interestingly, despite no actual quantitative improvements in TST or SE with Pod ON (vs. OFF), subjects perceived their TST, SE, SOL, sleep quality, and daytime dysfunction improved with Pod ON (assessed via PSQI; see Table 3).

This disparity between physiological sleep quality and perceptual sleep quality raises the interesting point that perceptions of sleep quality may not always match reality [77]. Future research could further test these mind-body connections by providing more educational content (via an Application) to subjects about their sleep the previous night. To evaluate the influence of education on perceived sleep quality, one could provide positive feedback about the subject’s or patient’s sleep despite actual physiological improvements, or alternately, provide negative feedback about their sleep when there are actual improvements. Such a study would tease apart the impact of education on the perception of sleep quality.

Lastly, previous research shows that thermal comfort and thermal sensation during sleep have an immense impact on sleep quality [18,19,37,38,59,65]. Both sexes reported feeling more comfortable with their body temperature during sleep with Pod ON vs. OFF, and that their body temperature felt cooler overall with Pod ON [Table 4]. As each person has different temperature needs based on their individual body temperature rhythm [21,26,51,52,53], the Pod allowed each individual to sleep at the temperature they found most comfortable. In turn, this led to subjects perceiving that it was easier to fall asleep and that their sleep was calmer [Table 5]. Subjects who were more comfortable with their body temperature also had more light sleep, TST, and higher SE [Table 5]. Thus, the Pod’s continuous temperature regulation improved subjects’ thermal comfort, leading to increased quantitative and qualitative sleep quality.

### 4.6. Limitations

A limitation of this study is the lack of skin and core temperatures to make conclusive claims about a causal relationship. From our results, it is clear that sleeping on a temperature-regulated bed can improve time spent in specific sleep stages and that these modifications are dependent on sex and Pod temperature. It is assumed that skin temperatures are modified as a result of cooler or warmer Pod temperatures, and that these skin temperature modifications led to the changes in sleep stage durations. However, further research should evaluate skin and core body temperatures while sleeping on the Pod to determine the mechanism behind the Pod. Another potential limitation is the fact that subjects were allowed to choose their own temperatures on the Pod vs. the researchers controlling subjects’ temperature settings. While controlling the temperature settings for all individuals would lead to a more controlled study, it would not account for variations in body temperature among the population. For example, putting both males and females at a 24 °C Pod temperature would not result in the same body temperature for everyone since each individual would be at a different body temperature before sleeping on the Pod. Thus, by allowing individuals to select their own temperatures, we could maximize ecological validity and mimic behavior typical of what people would do in the real world. A future study controlling Pod temperature while measuring core and skin temperatures throughout the night would likely uncover the true mechanism behind how the Pod modifies sleep. Lastly, measuring sleep stages with full polysomnography (vs. an HST) may further elucidate how sleep is modified with the Pod when sleeping at cool vs. warm temperatures.

Another potential limitation was the lack of controlled bedroom environmental conditions. Although we did ask subjects to keep the same bedroom conditions throughout the study, we did not measure ambient temperature and humidity and, therefore, do not definitively know whether these variables may have played a role in the study outcomes. That said, even without controlling for bedroom environmental conditions, we saw significant improvements in sleep and cardiovascular recovery while people slept with Pod ON, which disappeared with Pod OFF again at the end of the week. Any confounding variables (like environmental temperature, humidity, etc.) would only make the study results less significant, and so the fact that we still had significant findings without controlling for subjects’ bedroom temperatures or room conditions means that our findings are robust to these confounding factors. It would be interesting to understand whether specific Pod temperatures could improve sleep in very hot or cold bedroom conditions, as a cooler Pod temperature on a hot night may help maintain optimal skin and core temperatures for sleep.

Finally, we did not control for the menstrual cycle or menopausal status in our study. However, previous research has shown that the menstrual cycle phase does not measurably affect sleep [80,81,82], so we do not expect that menstrual cycle status played a role in our findings. Moreover, only 3 out of 27 women were menopausal, so it is unlikely these few subjects affected our findings—especially since each individual was compared to themselves over time.

## 5. Conclusions

By having subjects alternate their bed temperatures off and on over a two-week period, we could assess the impacts of a temperature-regulated mattress cover (the Eight Sleep Pod) on sleep and cardiovascular metrics, as well as subjects’ perceptions of their sleep. To our knowledge, this is the first study to show that manipulating the sleeping surface temperature during particular parts of the night can optimize deep, light, and REM sleep stages, and improve cardiovascular recovery by decreasing HR and increasing HRV. Sleeping at cooler temperatures in the first half of the night significantly increased deep sleep in men and REM sleep in women. Men sleeping at warmer temperatures during the second half of the night obtained more light sleep compared to those with no temperature regulation. These changes led to a more balanced sleep architecture and significant improvements in thermal comfort, perceived sleep quality, and sleep satisfaction. As sleeping on a continuously temperature-regulated mattress cover improves qualitative and quantitative sleep quality, it can be used as a non-invasive, non-pharmacologic alternative for improving sleep quality and reducing sleep aid use in the 70 million-plus Americans with sleep disorders [7].

## Figures and Tables

**Table 1:** Population characteristics stratified by sex.

Variable	Total *(n* = 54)	Male (*n* = 27)	Female (*n* = 27)	
	Mean ± SD (% of n)	Mean ± SD (% of n)	Mean ± SD (% of n)	*p*-Value
Age (mean)	36.0 ± 14.4	38.3 ± 14.7	33.7 ± 13.9	0.241
Reported use of sleep medication	9 (16.7%)	2 (7.4%)	7 (25.9%)	0.085
Heart conditions	1 (1.9%)	1 (3.7%)	0 (0%)	0.997
Respiratory conditions	7 (13.0%)	4 (14.8%)	3 (11.1%)	0.191
Global PSQI (mean)	5.0 ± 2.3	4.3 ± 2.3	5.2 ± 2.2	**0.002**

Note: subjects were excluded if they were taking heart medications, had a pacemaker, or severe sleep apnea (see Section 2). Heart and respiratory conditions listed here included chronic asthma, sleep apnea (AHI < 30), and arrhythmia. The *p*-value indicates whether there was a statistical difference between men and women; bolded values indicate a statistical difference at *p* < 0.05.

**Table 3:** Odds ratios and 95% confidence intervals evaluating changes in responses to PSQI from Pod OFF to Pod ON overall.

Variable	Odds Ratio	SE	95% CI	z-Value	*p*-Value
Component 1 (Duration)	0.362	0.336	(0.187,0.700)	−3.023	**0.003**
Component 3 (Latency)	0.232	0.229	(0.148, 0.363)	−6.378	**<0.001**
Component 4 (Dysfunction During Day)	0.203	0.341	(0.104, 0.397)	−4.673	**<0.001**
Component 5 (Efficiency)	0.976	0.224	(0.629, 1.516)	−0.107	0.915
Component 6 (Quality)	0.120	0.377	(0.057, 0.251)	−5.622	**<0.001**
Component 7 (Medication)	0.224	0.300	(0.124, 0.402)	−4.987	**<0.001**

Note: Bolded values indicate significance at *p* < 0.05. PSQI: Pittsburg Sleep Quality Index (see Section 2 for details); SE: standard error; 95% CI: 95% confidence intervals.

**Table 4:** Odds ratios and 95% confidence intervals for changes in responses to daily perceptual questions from Pod OFF to Pod ON.

Variable	Odds Ratio	SE	95% CI	z-Value	*p*-Value
How would you rate the calmness of your sleep last night?	1.289	0.123	(1.013, 1.640)	2.063	**0.039**
How easy was it to fall asleep last night?	1.357	0.127	(1.059, 1.739)	2.41	**0.016**
How easy was it to wake up this morning?	1.144	0.125	(0.895, 1.461)	1.076	0.282
How refreshed do you feel after waking?	1.091	0.123	(0.859, 1.386)	0.712	0.477
How satisfied are you with your sleep last night?	1.249	0.121	(0.985, 1.583)	1.838	0.066
On average, throughout the night, what was your thermal sensation?	1.780	0.122	(1.401, 2.260)	4.726	**<0.001**
On average, how comfortable were you with your body temperature throughout the night?	1.803	0.148	(1.349, 2.410)	3.981	**<0.001**

Note: Bolded values indicate significance at *p* < 0.05. SE: standard error; 95% CI: 95% confidence intervals.

**Table 5:** Odds ratios and 95% confidence intervals to indicate how improvements in sleep metrics from Pod OFF to Pod ON affect responses to perceptual questions.

Variable	Odds Ratio	SE	95% CI	z-Value	*p*-Value
**How would you rate the calmness of your sleep last night?**					
Deep Sleep	1.061	0.204	(0.711, 1.583)	0.291	0.771
REM Sleep	1.178	0.200	(0.796, 1.744)	0.818	0.413
Light Sleep	1.164	0.203	(0.781, 1.732)	0.747	0.455
Wake	1.694	0.216	(1.109, 2.586)	2.441	**0.015**
Total Sleep Time	1.297	0.220	(0.843, 1.996)	1.183	0.237
Number of Awakenings	1.037	0.208	(0.690, 1.555)	0.169	0.866
REM Sleep Onset	0.924	0.205	(0.617, 1.382)	−0.169	0.699
Deep Sleep Onset	0.942	0.222	(0.610, 1.457)	−0.267	0.790
Sleep Efficiency	1.358	0.129	(1.050, 1.750)	2.365	**0.018**
**How easy was it to fall asleep last night?**					
Deep Sleep	0.887	0.223	(0.573, 1.375)	−0.535	0.592
REM Sleep	1.434	0.221	(0.930, 2.212)	1.631	0.103
Light Sleep	0.995	0.221	(0.645, 1.535)	−0.023	0.981
Wake	2.027	0.264	(1.208, 3.400)	−2.676	**0.007**
Total Sleep Time	1.248	0.702	(0.315, 4.941)	0.316	0.752
Number of Awakenings	1.438	0.234	(0.908, 2.276)	1.549	0.121
Sleep Onset	1.246	0.232	(0.791, 1.962)	0.950	0.342
REM Sleep Onset	0.491	0.225	(0.316, 0.763)	−3.160	**0.002**
Deep Sleep Onset	0.815	0.240	(0.509, 1.303)	−0.855	0.393
Sleep Efficiency	1.557	0.136	(1.194, 2.030)	3.267	**0.001**
**How easy was it to wake up this morning?**					
Deep Sleep	0.842	0.206	(0.562, 1.262)	−0.832	0.406
REM Sleep	1.275	0.200	(0.863, 1.885)	1.219	0.223
Light Sleep	1.208	0.205	(0.809, 1.804)	0.925	0.355
Wake	1.247	0.220	(0.521, 1.236)	1.000	0.317
Total Sleep Time	1.558	0.216	(1.020, 2.380)	2.050	**0.040**
Number of Awakenings	1.158	0.211	(0.766, 1.750)	0.695	0.487
REM Sleep Onset	0.656	0.206	(0.438, 0.982)	−2.048	**0.041**
Deep Sleep Onset	1.083	0.222	(0.701, 1.674)	0.360	0.719
Sleep Efficiency	1.173	0.132	(0.906, 1.521)	1.210	0.226
**How refreshed do you feel after waking?**					
Deep Sleep	1.177	0.214	(0.761, 1.788)	0.761	0.447
REM Sleep	1.436	0.205	(0.960, 2.149)	1.762	0.078
Light Sleep	1.434	0.208	(0.953, 2.158)	1.730	0.084
Wake	1.578	0.238	(0.990, 2.514)	1.917	0.055
Total Sleep Time	1.561	0.226	(1.002, 2.433)	1.968	**0.049**
Number of Awakenings	0.898	0.217	(0.587, 1.375)	−0.492	0.623
REM Sleep Onset	0.771	0.213	(0.508, 1.171)	−1.219	0.223
Deep Sleep Onset	1.356	0.244	(0.841, 2.187)	1.249	0.212
Sleep Efficiency	1.442	0.366	(1.116, 1.865)	2.796	**0.005**
**How satisfied are you with your sleep last night?**					
Deep Sleep	1.129	0.204	(0.757, 1.683)	0.595	0.552
REM Sleep	1.512	0.212	(1.022, 2.235)	2.071	**0.038**
Light Sleep	1.28	0.201	(0.832, 1.899)	1.225	0.220
Wake	1.653	0.226	(1.061, 2.575)	2.223	**0.026**
Total Sleep Time	1.722	0.213	(1.135, 2.612)	2.555	**0.011**
Number of Awakenings	0.875	0.208	(0.582, 1.317)	−0.639	0.523
REM Sleep Onset	0.846	0.206	(0.564, 1.269)	−0.808	0.419
Deep Sleep Onset	1.381	0.233	(0.875, 2.179)	1.388	0.165
Sleep Efficiency	1.518	0.130	(1.178, 1.957)	3.222	**0.001**
**On average, throughout the night, what was your thermal sensation?**					
Deep Sleep	1.083	0.149	(0.809, 1.456)	0.537	0.591
REM Sleep	0.856	0.150	(0.635, 1.146)	−1.035	0.301
Light Sleep	0.687	0.156	(0.501, 0.927)	−2.400	**0.016**
Wake	1.038	0.149	(0.775, 1.039)	0.252	0.801
Total Sleep Time	0.759	0.163	(0.547, 1.041)	−1.693	0.091
Number of Awakenings	1.590	0.161	(1.171, 2.205)	−2.891	**0.004**
REM Sleep Onset	1.031	0.149	(0.767, 1.385)	0.201	0.841
Deep Sleep Onset	0.608	0.174	(0.425, 0.845)	−2.854	**0.004**
Sleep Efficiency	0.922	0.101	(0.756, 1.123)	−0.805	0.421
**On average, how comfortable were you with your body temperature throughout the night?**					
Deep Sleep	1.686	0.347	(0.875, 3.458)	1.507	0.132
REM Sleep	1.556	0.344	(0.810, 3.172)	1.286	0.198
Light Sleep	2.361	0.379	(1.171, 5.220)	2.267	**0.023**
Wake	2.656	0.384	(1.306, 5.920)	2.547	**0.011**
Total Sleep Time	2.265	0.375	(1.127, 4.945)	2.183	**0.029**
Number of Awakenings	0.643	0.344	(0.315, 1.235)	−1.286	0.198
REM Sleep Onset	0.576	0.349	(0.279, 1.116)	−1.58	0.114
Deep Sleep Onset	1.318	0.381	(0.647, 2.949)	0.727	0.467
Sleep Efficiency	1.831	0.196	(1.255, 2.713)	3.084	**0.002**

Note: Improvement in sleep is indicated by an increase in minutes from Pod OFF Baseline to Pod ON in deep sleep, REM sleep, light sleep, total sleep time, REM sleep onset, deep sleep onset latency, or sleep efficiency (see Section 2.5 for details). Improvements in the number of awakenings and minutes of wake time were indicated by a decrease in these sleep metrics. Bolded values indicate significance at *p* < 0.05; SE: standard error; 95% CI: 95% confidence intervals.

## Data Availability

The underlying code and datasets for this study are not publicly available for proprietary reasons.

## Supplementary Materials

The following supporting information can be downloaded at: https://www.mdpi.com/article/10.3390/bioengineering11040352/s1, Table S1: Means ± SD of the Pod temperatures selected by subjects during the study while Pod temperatures were ON; Table S2: Mean change in HR and HRV comparing Pod OFF Baseline vs. Pod ON or Pod OFF End; Table S3: Mean change in exercise metrics comparing Pod ON to Pod OFF as reference; Table S4: Changes in HR and HRV associated with sleeping at warm and cool Phase temperatures compared to Pod OFF from models run separately in females and males; Table S5: Odds ratios and 95% confidence intervals evaluating the difference in PSQI responses comparing sleeping with Pod OFF to sleeping at cool and warm temperatures from models, run separately in females and males; Table S6: Odds ratios and 95% confidence intervals evaluating the difference in perceptual responses comparing sleeping with Pod OFF to sleeping at cool and warm temperatures from models ran separately in females and males; Table S7: Odds ratios and 95% confidence intervals showing how changes in sleep metrics from Pod OFF to Pod ON affect responses to perceptual questions from models run separately in females and males; Table S8: Baseline (Pod OFF) sleep and cardiovascular metrics split by sex; Figure S1: Picture of the Eight Sleep Pod and Hub; Figure S2: Distribution of responses to the daily perceptual questions while Pod ON, stratified by sex.
