# Supplementary material for: Sleeping for One Week on a Temperature-Controlled Mattress Cover Improves Sleep and Cardiovascular Recovery
Source: Bioengineering (Basel). 2024 Apr 3;11(4):352. doi: 10.3390/bioengineering11040352 (PMC11048088; doi:10.3390/bioengineering11040352)
Supplement: Supplementary file 1 [file bioengineering-11-00352-s001.zip › bioengineering-2877469-supplementary.pdf]

Supplemental Information (SI)

Details on Fitbit Exercise Zones

For each exercise session that was logged by a subject or auto-detected by Fitbit, each exercise minute was categorized into a heart rate zone based on the heart rate for that minute as a percentage of the subject's age-predicted maximum HR. Subjects were asked to enter their demographic information (age, weight, height, and sex) in the Fitbit application at the beginning of the study to ensure accurate classification of HR zones based on age-predicted maximum heart rate. Fitbit classifies fat burn, cardio, and peak heart rate zones as 40–59%, 60–84%, and >85% of maximum HR, respectively.

Additional Details on Filtering Methods for HST and Fitbit

Fitbit sleep sessions with a duration of less than three hours were removed from the dataset because Fitbit does not report HR, HRV, or sleep staging for sessions under 3 h[48]. Subjects were included in the Fitbit analysis (n = 54) only if they had at least four out of seven nights for Pod OFF Baseline, five out of seven nights for Pod ON beginning and Pod ON end combined, and one out of the two nights for Post-Pod OFF. After applying these filtering criteria, HST sleep sessions with TST <3 h (~7% of nights) were removed from the dataset to match the Fitbit cutoff of 3 h.

Since there were several HST nights, with a significant portion of the night containing the "?" stage (due to loose electrodes or noisy EEG data), there was further filtering to ensure that only nights with reliable sleep staging were kept. For each night, we calculated the longest time period of consecutive minutes with a "?" stage. This highest count of missing data for a given night was divided by the total duration (including sleep and wake time) of the HST recording to define a percentage of missing ("?") data. Any night with >16% missing data was removed from the dataset. The 16% threshold was determined by visually examining the percent of time spent in deep and REM stages. In evaluating

different thresholds for the missing data cutoff, we found that there was little difference in the aggregate statistics for the percentage of time spent in deep and REM stages by filtering out nights with >10% vs. >16% missing data. However, the nights with greater than 16% missing data had significant differences in time spent in deep and REM stages when compared to Fitbit's sleep staging percentages. After all these filtering steps, subjects were only included in the final analyses if they had at least one HST night in each of the four temperature status categories (listed above).

Figures and Tables

Table S1. Means ± SD of the Pod temperatures selected by subjects during the study while Pod temperatures were ON.

Temperature Phase	Female	Male
**Bedtime Phase**	-1.0 ± 3.3	-3.1 ± 3.4
**Early Phase**	-1.0 ± 2.1	-2.0 ± 2.2
**Late Phase**	-0.1 ± 1.8	-0.7 ± 2.1

Note: Women were recommended -1, 0, and +1, while men were recommended -2, -1, and 0, which correspond to water temperatures of approximately 26, 27, and 29°C vs. 25, 26, and 27°C, respectively. On average, women and men modified these temperatures by -1 in the Early Phase and Late Phase, and men modified the Bedtime Phase temperature by -1.

Table S2. Mean change in HR and HRV comparing Pod OFF Baseline vs. Pod ON or Pod OFF End.

Variable	Change	Std Err	95% CI	t score	p-value
Minimum Sleeping Heart Rate					
Pod ON	-1.173	0.278	(-1.718, -0.628)	-4.218	<0.001
Pod OFF End	-0.383	0.405	(-1.178, 0.413)	-0.943	0.346
Median Sleeping HRV					
Pod ON	1.985	0.768	(0.481, 3.490)	2.587	0.009
Pod OFF End	0.566	1.114	(-1.617, 2.749)	0.508	0.611

Table S3. Mean change in exercise metrics comparing Pod ON to Pod OFF as reference.

Variable	Change	Std Err	95% CI	t value	p-value
Cardio (Min)	-0.638	0.657	(-1.927, 0.650)	5.020	0.332
Fat Burn (Min)	-0.044	2.081	(-4.126, 4.036)	-0.021	0.983
Steps (#)	-43.95	302.76	(-637.819, 549.685)	-0.145	0.885

Table S4. Changes in HR and HRV associated with sleeping at warm and cool Phase temperatures compared to Pod OFF from models ran separately in females and males

	Female					Male				
Variable	Change	Std Err	95% CI	t-value	p-value	Change	Std Err	95% CI	t-value	p-value
Minimum Sleeping Heart Rate										
Bedtime										
Cool	-0.119	0.575	(-1.244, 1.010)	-0.207	0.836	-1.45	0.607	(-2.639, -0.262)	-2.391	0.018
Warm	0.268	0.452	(-0.620, 1.153)	0.593	0.553	-0.837	0.473	(-1.763, 0.090)	-1.77	0.078
Early Phase										
Cool	0.148	0.503	(-0.837, 1.33)	0.293	0.769	-1.233	0.526	(-2.261, -0.200)	-2.345	0.02
Warm	0.133	0.498	(-0.844, 1.107)	0.267	0.79	-0.872	0.519	(-1.892, 0.144)	1.68	0.094
Late Phase										
Cool	0.416	0.488	(-0.541, 1.371)	0.852	0.395	-1.059	0.529	(-2.095, -0.024)	-2.003	0.046
Warm	-0.332	0.499	(-1.311, 0.646)	-0.665	0.507	-1.024	0.509	(-2.021, -0.027)	-2.013	0.045
Median Sleeping HRV										
Bedtime										
Cool	-1.877	1.977	(-5.747, 2.003)	-0.949	0.343	3.188	1.6	(0.053, 6.324)	1.992	0.047
Warm	0.195	1.554	(-2.854, 3.238)	0.125	0.901	0.049	1.244	(-2.388, 2.487)	0.039	0.969
Early Phase										
Cool	-3.096	1.718	(-6.457, 0.277)	-1.802	0.073	2.87	1.382	(0.163, 5.582)	2.076	0.039
Warm	1.958	1.702	(-1.382, 5.288)	1.515	0.251	-0.498	1.366	(-3.178, 2.176)	-0.365	0.716
Late Phase										
Cool	-1.721	1.684	(-5.016, 1.585)	-1.022	0.308	1.183	1.407	(-1.569, 3.944)	0.841	0.401
Warm	1.319	1.725	(-2.067, 4.693)	0.764	0.445	0.936	1.353	(-1.719, 3.545)	0.692	0.490

Table S5. Odds ratios and 95% confidence intervals evaluating the difference in PSQI responses comparing sleeping with pod OFF to sleeping at cool and warm temperatures from models ran separately in females and males.

	Female					Male				
Variable	Odds Ratio	Std Err	95% CI	z value	p-value	Odds Ratio	Std Err	95% CI	z value	p-value
**Component 1 (Duration)**										
Bedtime										
Cool	1.62	0.791	(0.344, 7.633)	0.609	0.542	1.083	0.787	(0.231, 5.065)	0.101	0.919
Warm	0.956	0.524	(0.343, 2.667)	-0.087	0.931	0.075	0.605	(0.023, 0.245)	-4.285	<0.001
Early Phase										
Cool	1.598	0.718	(0.392, 6.524)	0.654	0.513	0.316	0.549	(0.108, 0.927)	-2.099	0.036
Warm	0.965	0.5	(0.362, 2.571)	-0.072	0.943	0.082	0.594	(0.026, 0.264)	-4.203	<0.001
Late Phase										
Cool	1.197	0.599	(0.370, 3.871)	0.301	0.764	0.201	0.517	(0.073, 0.553)	-3.107	0.002
Warm	1.031	0.525	(0.368, 2.884)	0.057	0.954	0.148	0.509	(0.055, 0.401)	-3.752	<0.001
**Component 3 (Latency)**										
Bedtime										
Cool	0.265	0.433	(0.113, 0.619)	-3.068	0.002	0.506	0.534	(0.174, 1.467)	-1.255	0.21
Warm	0.177	0.351	(0.089, 0.352)	-4.933	<0.001	0.203	0.39	(0.094, 0.435)	-4.099	<0.001
Early Phase										
Cool	0.276	0.382	(0.130, 0.583)	-3.37	0.001	0.373	0.412	(0.166, 0.837)	-2.39	0.017
Warm	0.154	0.386	(0.072, 0.329)	-4.848	<0.001	0.188	0.446	(0.078, 0.450)	-3.753	<0.001
Late Phase										
Cool	0.336	0.356	(0.167, 0.676)	-3.06	0.002	0.255	0.421	(0.112, 0.582)	-3.245	0.001
Warm	0.112	0.426	(0.049, 0.259)	-5.13	<0.001	0.286	0.422	(0.125, 0.655)	-2.962	0.003
**Component 4 (Dysfunction During Day)**										
Bedtime										
Cool	0.151	0.592	(0.047, 0.482)	-3.195	0.001	0.003	1677.7	(0.000, Inf)	-0.005	0.996
Warm	0.331	0.414	(0.147, 0.745)	-2.67	0.007	0.146	0.723	(0.035, 0.602)	-2.662	0.008
Early Phase										
Cool	0.236	0.443	(0.099, 0.564)	-3.253	0.001	0.003	593.16	(0.000, Inf)	-0.01	0.992
Warm	0.255	0.452	(0.105, 0.627)	-3.031	0.002	0.533	0.609	(0.180, 1.959)	-0.857	0.391
Late Phase										
Cool	0.185	0.465	(0.075, 0.461)	-3.626	<0.001	0.226	0.556	(0.076, 0.672)	-2.674	0.008
Warm	0.323	0.432	(0.139, 0.754)	-2.615	0.009	0.132	0.629	(0.038, 0.451)	-3.226	0.001
**Component 5 (Efficiency)**										
Bedtime										
Cool	2.128	0.442	(0.895, 5.056)	1.709	0.087	2.362	0.493	(0.899, 6.204)	1.745	0.081
Warm	1.807	0.369	(0.876, 3.725)	1.602	0.109	0.34	0.361	(0.168, 0.691)	-2.985	0.003
Early Phase										
Cool	2.183	0.431	(0.938, 5.082)	1.812	0.07	1.104	0.371	(0.534, 2.288)	0.269	0.788
Warm	1.761	0.376	(0.843, 3.681)	1.505	0.132	0.403	0.348	(0.204, 0.797)	-2.61	0.009
Late Phase										
Cool	1.847	0.386	(0.867, 3.936)	1.591	0.112	1.04	0.369	(0.504, 2.148)	0.107	0.915
Warm	1.9	0.372	(0.918, 3.936)	1.728	0.084	0.409	0.36	(0.202, 0.828)	-2.485	0.013
**Component 6 (Quality)**										
Bedtime										
Cool	0.176	0.666	(0.048, 0.650)	-2.606	0.009	0.012	1.266	(0.001, 0.143)	-3.498	<0.014
Warm	0.082	0.562	(0.027, 0.247)	-4.447	<0.001	0.138	0.598	(0.043, 0.447)	-3.309	0.001
Early Phase										
Cool	0.162	0.544	(0.056, 0.471)	-3.343	0.001	0.085	0.671	(0.023, 0.317)	-3.671	0.001

Warm	0.051	0.714	(0.013, 0.207)	-4.164	<0.001	0.155	0.561	(0.051, 0.464)	-3.326	0.008
Late Phase										
Cool	0.072	0.682	(0.019, 0.274)	-3.859	<0.001	0.094	0.596	(0.029, 0.301)	-3.974	<0.001
Warm	0.128	0.524	(0.046, 0.59)	-3.916	<0.001	0.17	0.563	(0.056, 0.511)	-3.15	0.002
**Component 7 (Medication)**										
Bedtime										
Cool	0.41	0.539	(0.142, 1.179)	-1.655	0.098	0.081	0.89	(0.014, 0.465)	-2.82	0.004
Warm	0.181	0.492	(0.069, 0.476)	-3.468	0.001	0.226	0.473	(0.090, 0.572)	-3.14	0.002
Early Phase										
Cool	0.239	0.528	(0.085, 0.672)	-2.714	0.007	0.071	0.671	(0.019, 0.265)	-3.938	<0.001
Warm	0.265	0.463	(0.107, 0.656)	-2.872	0.004	0.41	0.484	(0.158, 1.058)	-1.843	0.065
Late Phase										
Cool	0.196	0.492	(0.075, 0.515)	-3.306	0.001	0.155	0.575	(0.050, 0.477)	-3.246	0.001
Warm	0.29	0.427	(0.125, 0.670)	-2.899	0.004	0.269	0.501	(0.101, 0.717)	-2.623	0.009

Table S6. Odds ratios and 95% confidence intervals evaluating the difference in perceptual responses comparing sleeping with pod OFF to sleeping at cool and warm temperatures from models ran separately in females and males.

	Female					Male				
Variable	Odds Ratio	Std Err	95% CI	z value	p-value	Odds Ratio	Std Err	95% CI	z value	p-value
**How would you rate the calmness of your sleep last night?**										
Bedtime										
Cool	1.136	0.182	(0.794, 1.624)	0.697	0.486	2.891	0.194	(1.975, 4.232)	5.459	<0.001
Warm	1.264	0.15	(0.942, 1.696)	1.565	0.118	1.499	0.15	(1.117, 2.011)	1.322	0.006
Early Phase										
Cool	1.162	0.163	(0.845, 1.598)	0.923	0.356	2.171	0.167	(1.564, 3.014)	4.635	<0.001
Warm	1.26	0.162	(0.920, 1.739)	1.449	0.147	1.677	0.164	(1.216, 2.313)	3.153	0.002
Late Phase										
Cool	1.278	0.161	(0.842, 1.582)	0.891	0.373	2.177	0.168	(1.566, 3.027)	4.624	<0.001
Warm	1.154	0.164	(0.926, 1.762)	1.493	0.135	1.629	0.163	(1.183, 2.244)	2.986	0.003
**How easy was it to fall asleep last night?**										
Bedtime										
Cool	1.294	0.194	(0.884, 1.892)	1.326	0.185	1.757	0.197	(1.194, 2.585)	2.86	0.004
Warm	1.744	0.163	(1.268, 2.400)	3.419	0.001	1.522	0.154	(1.125, 2.059)	2.724	0.006
Early Phase										
Cool	1.335	0.17	(0.957, 1.861)	1.701	0.089	1.686	0.17	(1.207, 2.355)	3.066	0.002
Warm	1.795	0.178	(1.266, 2.545)	3.286	0.001	1.516	0.168	(1.091, 2.105)	2.479	0.013
Late Phase										
Cool	1.281	0.168	(0.922, 1.779)	1.478	0.139	1.661	0.171	(1.188, 2.324)	2.964	0.003
Warm	1.803	0.179	(1.268, 2.562)	3.285	0.001	1.498	0.167	(1.080, 2.078)	2.418	0.016
**How easy was it to wake up this morning?**										
Bedtime										
Cool	1.264	0.194	(0.865, 1.847)	1.21	0.226	1.255	0.186	(0.873, 1.806)	1.227	0.22
Warm	1.046	0.157	(0.769, 1.421)	0.286	0.775	1.116	0.147	(0.837, 1.490)	0.748	0.454
Early Phase										
Cool	1.042	0.168	(0.749, 1.449)	0.242	0.809	1.233	0.163	(0.896, 1.696)	1.288	0.198
Warm	1.222	0.172	(0.872,1.712)	1.163	0.245	1.112	0.159	(0.815, 1.517)	0.67	0.503
Late Phase										
Cool	1.219	0.168	(0.877, 1.693)	1.18	0.238	1.177	0.164	(0.854, 1.622)	0.995	0.32
Warm	1.182	0.173	(0.842, 1.659)	0.964	0.335	1.204	0.161	(0.879, 1.649)	1.157	0.247
**How refreshed do you feel after waking?**										
Bedtime										

Cool	1.258	0.185	(0.875, 1.807)	1.24	0.215	1.41	0.188	(0.975, 2.040)	1.827	0.068
Warm	1.352	0.151	(1.006, 1.817)	1.999	0.046	1.207	0.149	(0.901, 1.618)	1.264	0.206
Early Phase										
Cool	1.153	0.164	(0.837, 1.589)	0.874	0.382	1.295	0.163	(0.941, 1.782)	1.589	0.112
Warm	1.478	0.164	(1.071, 2.039)	2.375	0.018	1.25	0.162	(0.911, 1.716)	1.384	0.166
Late Phase										
Cool	1.138	0.162	(0.828, 1.564)	0.796	0.426	1.268	0.164	(0.919, 1.750)	1.446	0.148
Warm	1.611	0.167	(1.161, 2.236)	2.855	0.004	1.33	0.162	(0.968, 1.827)	1.761	0.078
**How satisfied are you with your sleep last night?**										
Bedtime										
Cool	1.301	0.179	(0.916, 1.847)	1.47	0.142	1.835	0.188	(1.270, 2.650)	3.236	0.001
Warm	1.45	0.148	(1.086, 1.936)	2.52	0.012	1.379	0.149	(1.031, 1.845)	2.166	0.03
Early Phase										
Cool	1.291	0.16	(0.943, 1.766)	1.595	0.111	1.622	0.163	(1.179, 2.231)	2.974	0.003
Warm	1.508	0.16	(1.102, 2.065)	2.566	0.01	1.428	0.16	(1.043, 1.965)	2.22	0.026
Late Phase										
Cool	1.374	0.159	(1.006, 1.877)	1.999	0.046	1.575	0.164	(1.142, 2.172)	2.769	0.006
Warm	1.471	0.161	(1.072, 2.017)	2.394	0.017	1.588	0.162	(1.155, 2.183)	2.847	0.004
**On average, throughout the night, what was your thermal sensation?**										
Bedtime										
Cool	2.935	0.183	(2.052, 4.120)	5.899	<0.001	5.109	0.199	(3.458, 7.550)	8.188	<0.001
Warm	1.115	0.145	(0.838, 1.482)	0.746	0.456	2.218	0.151	(1.650, 2.983)	5.271	<0.001
Early Phase										
Cool	2.391	0.164	(1.734, 3.297)	5.318	<0.001	4.384	0.172	(3.130, 6.140)	8.6	<0.001
Warm	1.092	0.158	(0.802, 1.487)	0.556	0.578	2.076	0.159	(1.520, 2.837)	4.588	<0.001
Late Phase										
Cool	2.076	0.161	(1.513, 2.848)	4.529	<0.001	4.217	0.174	(2.997, 5.934)	8.259	<0.001
Warm	1.137	0.158	(0.835, 1.550)	0.815	0.415	2.135	0.161	(1.558, 2.925)	4.722	<0.001
**On average, how comfortable were you with your body temperature throughout the night?**										
Bedtime										
Cool	1.546	0.208	(1.028, 2.324)	2.093	0.036	6.66	0.909	(1.122, 39.539)	2.087	0.037
Warm	1.587	0.174	(1.128, 2.232)	2.652	0.008	1.915	0.286	(1.094, 3.352)	2.276	0.023
Early Phase										
Cool	1.399	0.183	(0.977, 2.002)	1.834	0.067	1.757	0.198	(1.757, 3.815)	4.81	<0.001
Warm	1.776	0.191	(1.222, 2.582)	3.009	0.003	1.331	0.181	(1.331, 2.711)	3.538	<0.001
Late Phase										
Cool	1.908	0.195	(1.303, 2.795)	3.319	<0.001	2.957	0.208	(1.969, 4.441)	5.224	<0.001
Warm	1.436	0.186	(0.997, 2.066)	1.946	0.052	1.73	0.183	(1.208, 2.477)	2.994	0.003

Table S7. Odds ratios and 95% confidence intervals showing how changes in sleep metrics from Pod OFF to Pod ON affect responses to perceptual questions, from models run separately in females and males.

Variable	Women					Men				
	Odds Ratio	Std Err	95% CI	z value	p-value	Odds Ratio	Std Err	95% CI	z value	p-value
How would you rate the calmness of your sleep last night?										
Deep Sleep	1.405	0.272	(0.825, 2.393)	1.252	0.211	0.699	0.313	(0.378, 1.293)	-1.142	0.254
REM Sleep	1.284	0.266	(0.762, 2.162)	0.939	0.348	1.092	0.305	(0.600, 1.984)	0.287	0.774
Light Sleep	1.265	0.272	(0.742, 2.155)	0.864	0.388	1.048	0.305	(0.576, 1.905)	0.153	0.878
Wake	0.712	0.296	(0.399, 1.271)	-1.149	0.251	0.503	0.324	(0.267, 0.950)	-2.119	0.034
Total Sleep Time	1.739	0.280	(1.004, 3.011)	1.976	0.048	2.077	0.326	(0.518, 1.860)	-0.055	0.956
Number of Awakenings	0.839	0.294	(0.472, 1.493)	-0.596	0.551	1.130	0.306	(0.620, 2.060)	0.400	0.689
REM Sleep Onset	1.070	0.283	(0.614, 1.865)	0.239	0.811	0.371	0.334	(0.193, 0.713	-2.971	0.003
Deep Sleep Onset	1.379	0.210	(0.796, 2.390)	1.145	0.252	0.580	0.341	(0.297, 1.132)	-1.597	0.110
Sleep Efficiency	1.316	0.184	(0.919, 1.887)	1.498	0.134	1.396	0.183	(0.975, 1.999)	1.822	0.068
How easy was it to fall asleep last night?										
Deep Sleep	1.252	0.310	(0.682, 2.297)	0.726	0.468	0.609	0.327	(0.321, 1.157)	-1.514	0.130
REM Sleep	1.171	0.297	(0.654, 2.094)	0.531	0.595	1.922	0.358	(0.954, 3.873)	1.828	0.068
Light Sleep	1.074	0.308	(0.586, 1.965)	0.230	0.818	0.915	0.322	(0.487, 1.719)	-0.276	0.782
Wake	0.600	0.373	(0.289, 1.245)	-1.371	0.170	0.389	0.382	(0.184, 0.822)	-2.473	0.013
Total Sleep Time	1.010	0.323	(0.541, 1.920)	0.057	0.954	0.690	0.342	(0.353, 1.350)	-1.083	0.279
Number of Awakenings	1.158	0.370	(0.561, 2.394)	0.397	0.691	1.923	0.319	(1.030, 3.590)	2.053	0.040
Sleep Onset	0.703	0.340	(0.361, 1.370)	0.340	0.301	2.281	0.351	(1.147, 4.534)	2.352	0.019
REM Sleep Onset	0.630	0.315	(0.339, 1.168)	-1.467	0.142	0.356	0.371	(0.172, 0.737)	-2.781	0.005
Deep Sleep Onset	1.102	0.314	(0.596, 2.038)	0.309	0.757	0.705	0.343	(0.361, 1.380)	-1.019	0.308
Sleep Efficiency	1.763	0.199	(1.194, 2.604)	2.851	0.004	1.412	0.002	(1.406, 1.417)	183.50	<0.001
How easy was it to wake up this morning?										
Deep Sleep	0.963	0.282	(0.554, 1.676)	-0.132	0.895	0.611	0.292	(0.344, 1.084)	-1.684	0.092
REM Sleep	1.312	0.274	(0.767, 2.243)	0.993	0.321	1.242	0.291	(0.702, 2.200)	0.745	0.456
Light Sleep	1.396	0.286	(0.796, 2.447)	1.165	0.244	1.147	0.310	(0.625, 2.105)	0.441	0.659
Wake	0.908	0.317	(0.488, 1.690)	-0.305	0.760	0.803	0.307	(0.439, 1.466)	-0.716	0.474
Total Sleep Time	2.224	0.296	(1.244, 3.974)	2.698	0.006	1.167	0.297	(0.652, 2.091)	0.521	0.603
Number of Awakenings	0.951	0.309	(0.519, 1.740)	-0.164	0.869	1.333	0.289	(0.756, 2.350)	0.995	0.320
REM Sleep Onset	0.659	0.295	(0.369, 1.175)	-1.415	0.157	0.438	0.325	(0.232, 0.827)	-2.545	0.011
Deep Sleep Onset	1.210	0.290	(0.685, 2.137)	0.656	0.512	0.695	0.341	(0.356, 1.354)	-1.070	0.284
Sleep Efficiency	1.233	0.187	(0.854, 1.780)	1.119	0.263	1.056	0.187	(0.732, 1.523)	0.291	0.771
How refreshed do you feel after waking?										
Deep Sleep	1.709	0.313	(0.926, 3.152)	1.714	0.087	0.822	0.310	(0.448, 1.509)	-0.631	0.528
REM Sleep	1.534	0.287	(0.874, 2.692)	1.492	0.136	1.433	0.302	(0.793, 2.588)	1.191	0.234
Light Sleep	1.351	0.302	(0.748, 2.439)	0.998	0.318	1.524	0.294	(0.856, 2.713)	1.430	0.153
Wake	0.690	0.334	(0.359, 1.327)	-1.111	0.266	0.616	0.361	(0.304, 1.249)	-1.344	0.179

Total Sleep Time	2.120	0.319	(1.135, 3.962)	2.357	0.018	1.389	0.318	(0.744, 2.591)	1.032	0.302
Number of Awakenings	0.826	0.322	(0.439, 1.553)	-0.595	0.552	0.884	0.302	(0.489, 1.597)	-0.409	0.683
REM Sleep Onset	0.838	0.310	(0.456, 1.538)	-0.571	0.568	0.374	0.333	(0.195, 0.719)	-2.953	0.003
Deep Sleep Onset	1.777	0.311	(0.965, 3.270)	1.847	0.065	0.870	0.360	(0.430, 1.761)	-0.386	0.700
Sleep Efficiency	1.663	0.190	(1.147, 2.413)	2.681	0.007	1.246	0.183	(0.870, 1.784)	1.202	0.229
How satisfied are you with your sleep last night?										
Deep Sleep	0.822	0.310	(0.448, 1.509)	-0.631	0.528	1.132	0.324	(0.600, 2.135)	0.383	0.701
REM Sleep	1.388	0.256	(0.840, 2.295)	1.279	0.201	1.869	0.326	(0.986, 3.543)	1.917	0.055
Light Sleep	1.049	0.263	(0.626, 1.758)	0.181	0.856	1.625	0.309	(0.887, 2.980)	1.571	0.116
Wake	0.582	0.303	(0.321, 1.055)	-1.783	0.075	0.729	0.347	(0.369, 1.438)	-0.912	0.362
Total Sleep Time	1.844	0.265	(1.097, 3.099)	2.309	0.021	1.769	0.331	(0.924, 3.387)	1.721	0.085
Number of Awakenings	0.720	0.273	(0.422, 1.229)	-1.205	0.228	0.921	0.317	(0.495, 1.715)	-0.259	0.795
REM Sleep Onset	0.852	0.271	(0.502, 1.448)	-0.591	0.555	0.422	0.339	(0.217, 0.820)	-2.544	0.011
Deep Sleep Onset	1.584	0.276	(0.923, 2.718)	1.669	0.095	0.884	0.377	(0.422, 1.850)	-0.328	0.743
Sleep Efficiency	1.705	0.184	(1.188, 2.445)	2.896	0.004	1.355	0.184	(0.946, 1.942)	1.657	0.098
On average, throughout the night, what was your thermal sensation?*										
Deep Sleep	0.790	0.206	(0.518, 1.172)	-1.148	0.251	1.565	0.247	(0.978, 2.598)	1.815	0.070
REM Sleep	0.981	0.200	(0.718, 1.590)	0.308	0.758	0.670	0.242	(0.409, 1.066)	-1.651	0.099
Light Sleep	0.532	0.239	(0.319, 0.824)	-2.644	0.008	0.845	0.233	(0.530, 1.332)	-0.724	0.469
Wake	1.130	0.203	(0.761, 1.707)	0.601	0.548	0.909	0.242	0.560, 1.459)	-0.394	0.693
Total Sleep Time	0.572	0.228	(0.353, 0.873)	-2.449	0.014	0.860	0.235	(0.537, 1.363)	-0.643	0.520
Number of Awakenings	1.350	0.207	(0.906, 2.063)	1.445	0.148	1.828	0.259	(1.124, 3.128)	2.334	0.020
REM Sleep Onset	1.102	0.204	(0.735, 1.657)	0.474	0.636	0.643	0.270	(0.368, 1.073)	-1.637	0.102
Deep Sleep Onset	0.759	0.208	(0.495, 1.129)	-1.326	0.185	0.588	0.264	(0.340, 0.970)	-2.005	0.045
Sleep Efficiency	0.838	0.146	(0.625, 1.114)	-1.206	0.228	1.001	0.141	(0.759, 1.322)	0.011	0.992
On average, how comfortable were you with your body temperature throughout the night?*										
Deep Sleep	2.608	0.534	(0.961, 8.035)	1.794	0.073	1.129	0.449	(0.445, 2.848)	0.271	0.787
REM Sleep	1.403	0.484	(0.542, 3.715)	0.700	0.484	1.934	0.550	(0.741, 6.878)	1.200	0.230
Light Sleep	1.642	0.497	(0.633, 4.554)	0.999	0.318	3.801	0.666	(1.237, 17.593	2.006	0.045
Wake	0.612	0.509	(0.212, 1.611)	-0.965	0.335	0.245	0.620	(0.062, 0.718)	-2.268	0.023
Total Sleep Time	1.995	0.500	(0.766, 5.576)	1.380	0.168	3.208	0.598	(1.144, 12.255)	1.951	0.051
Number of Awakenings	0.741	0.485	(0.282, 1.940)	-0.619	0.536	0.425	0.626	(0.097, 1.210)	-1.367	0.172
REM Sleep Onset	0.974	0.499	(0.368, 2.691)	-0.053	0.958	0.350	0.536	(0.108, 0.917)	-1.960	0.050
Deep Sleep Onset	1.698	0.498	(0.653, 4.725)	1.063	0.288	0.949	0.507	(0.338, 2.877)	-0.102	0.919
Sleep Efficiency	1.959	0.305	(1.092, 3.634)	2.205	0.028	1.772	0.256	(1.084. 2.979)	2.235	0.025

Table S8. Baseline (Pod OFF) sleep and cardiovascular metrics split by sex.

	**Men (mean ± SD)**	**Women (mean ± SD)**	***p*-value**
Deep (min)	83.24±32.60	97.18±53.92	0.252
Deep (%)	22.24±8.92	24.24±13.06	0.497
REM (min)	78.80±47.93	65.91±38.58	0.181
REM (%)	19.75±10.48	16.52±9.58	0.155
Wake (min)	39.37±19.54	39.96±21.77	0.929
Light (min)	221.1±57.64	239.2±72.86	0.280
Light (%)	58.24±12.08	59.24±15.11	0.592
HR	58.93±10.45	64.99±6.47	**0.016**
HRV	51.65±27.93	45.17±25.60	0.319
Number of awakenings	30.14±11.04	27.95±7.09	0.422
REM Sleep Latency	141.20±83.74	136.3±97.81	0.812
Deep Sleep Latency	15.6±19.3	15.7±38.07	0.587
Sleep Efficiency	0.85±0.06	0.86±0.06	0.692
SOL (min)	35.13±23.49	39.59±28.48	0.785
TST (h)	6.39±1.24	6.71±0.95	0.342

Note: these data represent baseline (i.e., Pod OFF Baseline) averages for men and women from latency of persistent sleep (LPS) to sleep end. The *p*-value is the statistical difference between men's and women's values.

Figure S1: Picture of the Eight Sleep Pod and Hub.

The Eight Sleep Pod cover fits over the mattress like a fitted sheet and connects to the accompanying Hub beside the bed, which has a water tank. The hub changes the water temperature of the Pod. See the Methods for more details.

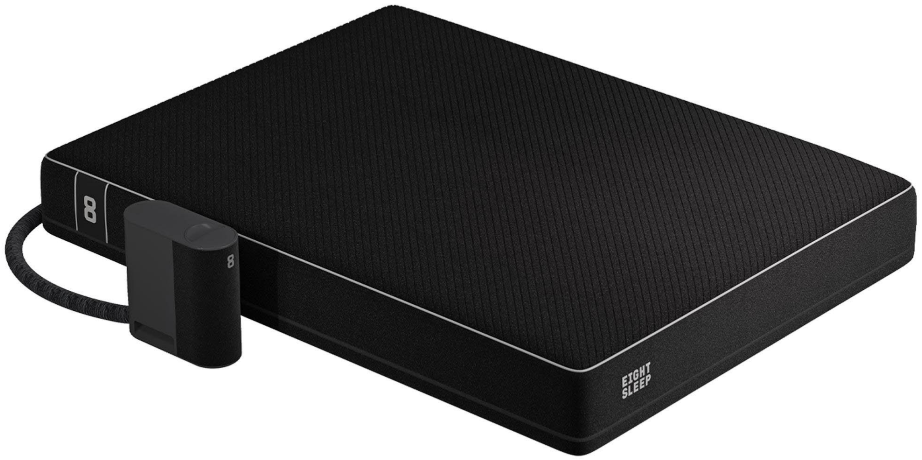

Figure S2: Distribution of responses to the daily perceptual questions while Pod ON, stratified by sex.

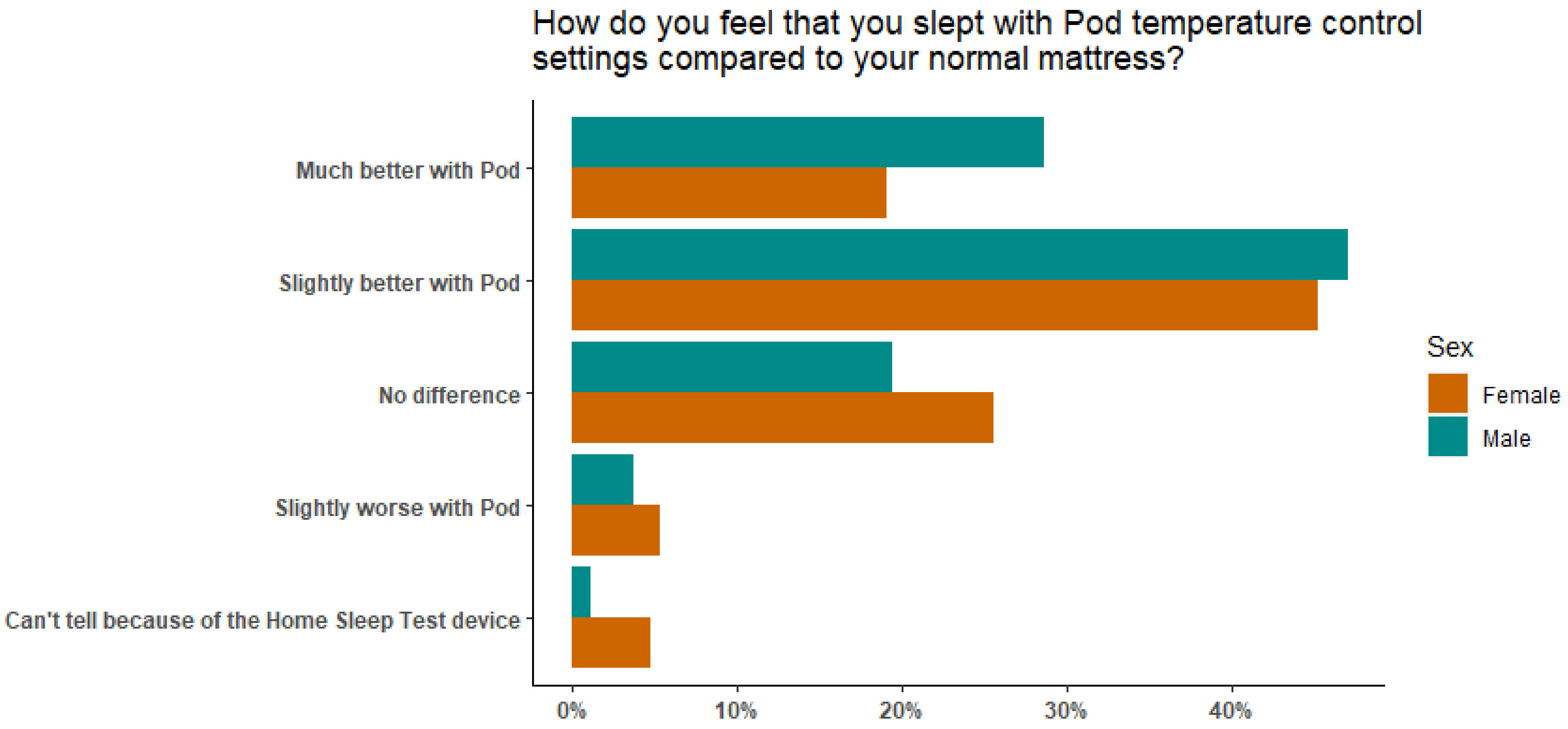

SA: Health History

The following questions are about your medical history. All questions will be kept confidential to the extent allowed by law and will be password-protected and secured. Please reach out to our team at rd.clinical@eightsleep.com with any questions.

1.) What is your Subject # for this study?
2.) What is your date of birth?
3.) What is your height (in inches)?
4.) What is your weight (in pounds)?
5.) How would you best describe yourself?
   ( ) American Indian or Alaska Native
   ( ) Asian
   ( ) Black or African American
   ( ) Hispanic, Latino, or Spanish
   ( ) Native Hawaiian or Other Pacific Islander
   ( ) White
   ( ) Other (Please specify)
   ( ) Prefer not to say.
6.) What is your biological sex? *Note that this is not the gender that you identify with.*
   ( ) male
   ( ) female
   ( ) other
7.) Which of the following best describes your current status? *Make between 1 and 3 choices.*
   ( ) regular menstrual cycle (every 28–32 days)—no birth control
   ( ) regular menstrual cycle—on some form of birth control
   ( ) irregular menstrual cycle—on some form of birth control
   ( ) irregular menstrual cycle—no birth control
   ( ) amenorrheic
   ( ) pre-menopausal with hormone replacement therapy
   ( ) pre-menopausal with no hormone replacement therapy
   ( ) pregnant
   ( ) breastfeeding
   ( ) post-menopausal with hormone replacement therapy
   ( ) post-menopausal—no hormone replacement therapy
   ( ) polycystic ovarian syndrome
   ( ) Other
8.) What gender do you most closely identify with?
   ( ) woman
   ( ) man
   ( ) transgender
   ( ) non-binary
   ( ) prefer not to say.
   ( ) Other

9.) What type of mattress do you sleep on?

( ) foam
( ) innerspring
( ) hybrid
( ) other

10.) What is the approximate thickness of your mattress (in inches)? *This is the measurement of how tall your mattress is when it is lying flat (as if you were to sleep on it)*

11.) What type of pillow do you sleep on?

( ) down
( ) synthetic down
( ) memory foam
( ) latex
( ) other

12.) How would you rate your pillow's firmness?

( ) soft
( ) medium
( ) firm

13.) What is the height/loft of your pillow? *This is a measure of the thickness of your pillow.*

( ) low loft
( ) medium loft
( ) high loft

14.) Do you have a child or pet that typically sleeps in the same bed as you?

( ) Yes
( ) No

15.) Please check all of the following conditions with which you have ever been diagnosed or for which you have been treated. *If you do not see your condition and/or illness, there will be another question that asks you to write it in*.

( ) high blood pressure
( ) chronic asthma
( ) coronary artery disease
( ) high cholesterol
( ) diabetes (Type I or II)
( ) kidney problems
( ) cardiovascular disease
( ) heart rhythm problems (e.g., atrial fibrillation, flutter, arrhythmia)
( ) chronic headaches
( ) anemia (sickle cell or otherwise)
( ) epilepsy (seizures)
( ) sleep apnea
( ) insomnia
( ) vascular problems (e.g., atherosclerosis)

( ) gastrointestinal disease (IBD, Chron's, etc.)
( ) bladder problems (frequent urination, etc.)
( ) lung disease (emphysema, COPD, chronic bronchitis, etc.)
( ) autoimmune disease (e.g., lupus, atopic dermatitis, etc.)
( ) stroke or hemorrhage
( ) restless leg syndrome
( ) periodic leg movement
( ) Raynaud's syndrome or peripheral artery disease
( ) narcolepsy
( ) None of the above

16.) Do you know your Apnea-Hypoxia Index (AHI) score?
( ) Yes
( ) No

17.) What is your AHI score?

18.) Please indicate what illness or condition you have below.

19.) Do you currently take any medications or have any conditions that alter your body temperature (e.g., lead to excessive sweating)? *Examples include ADHD medication, hot flashes for menopausal women, etc.*
( ) Yes
( ) No

20.) Do you currently take any medications that affect your heart rate? *This includes beta-blockers, alpha-blockers, ACE inhibitors, etc*.
( ) Yes
( ) No

21.) Do you currently take any medications for high blood pressure? *E.g., diuretics, ACE inhibitors, beta-blockers, etc.*
( ) Yes
( ) No

22.) Please list all medications you are currently taking. Make sure to include prescriptions for over-the-counter medication, supplements, or hormonal replacement/birth control. *Please write NA if you are not taking any.*

23.) Have you ever been hospitalized for a heart attack?
( ) Yes
( ) No

24.) Do you currently have a stent placed, or have you had an arterial bypass?
( ) Yes
( ) No

25.) Do you currently have a pacemaker or ICD implanted?
( ) Yes
( ) No

26.) Are you currently experiencing any respiratory issues? (e.g., cough, chest, congestion, runny or stuffy nose, sinus problems) *Please include any issues due to allergies.*

( ) Yes
( ) No

27.) Have you ever been told by your doctor that you are overweight or obese?
( ) Yes
( ) No

28.) Do you currently smoke cigarettes, vape, or cigars?
( ) Yes
( ) No

29.) How many years have you smoked?

30.) On average, how many cigarettes or cigars do you smoke per day? Or how many times do you vape per day? *If you only smoke 1 cigar per week, for example, please just write what the average would be per day (e.g., 1/7)*

31.) Did you previously smoke but have to quit?
( ) Yes
( ) No

32.) How many years did you previously smoke? *Note that these are the number of years you consistently smoked; if you had years that you quit smoking but started again, please include the total number of years you actually smoked (and exclude the years you didn't smoke).*

33.) Do you currently take any sleep medications or supplements? *Please include prescription as well as over-the-counter medications or supplements like melatonin*.
( ) Yes
( ) No

34.) What are the sleep medications or supplements that you take?

35.) On average, how many nights per week do you take this medication or supplement to help you sleep?

36.) On average, how many alcoholic drinks do you have per week?
( ) 0
( ) 1-7
( ) 8-14
( ) 14+

37.) Do you drink or take caffeine on a daily basis?
( ) Yes
( ) No

38.) In the last 2 weeks, how many days per week, on average, did you exercise for at least 20 mins (where you were sweating)?
( ) 0
( ) 1-2
( ) 3-5
( ) 6+

39.) Which of the following best describes your habits over the last year?
( ) Avid exerciser: 6-7 days per week for 30+ minutes each session
( ) Regular exerciser: 3-5 days per week for 30+ minutes each session

( ) Physically active: I do not intentionally exercise, but I move a lot throughout the day and/or have a physically intensive day job (e.g., construction worker)
( ) I am just beginning an exercise program now, but I plan to become a regular exerciser.
( ) I am an inconsistent exerciser: I fit in exercise when I have time, but it varies from week to week based on my priorities.
( ) I do not exercise.

40.) How would you rate your fitness as compared to people of your same age and sex?
( ) Above average fitness compared to people of my age and sex.
( ) Similar fitness compared to people of my age and sex.
( ) Below average fitness compared to people of my age and sex.

SB: pre-Pod PSQI

The following 10 questions are to begin building your sleep baseline.

These questions relate to your usual sleep habits during the past month only. Your answers should indicate the most accurate reply for the majority of the days and nights in the past month. Please answer all questions.

1.) During the past month, when have you usually gone to bed at night?
  a.) USUAL BEDTIME:
2.) During the past month, how long (IN MINUTES) has it usually taken you to fall asleep each night?
  a.) NUMBER OF MINUTES:
3.) During the past month, when have you usually gotten up in the morning
  a.) USUAL GETTING UP TIME:
4.) During the past month, how many hours of actual sleep did you get a night? (This may be different than the number of hours you spend in bed.)
  a.) HOURS OF SLEEP PER NIGHT
5.) During the past month, how would you rate your sleep quality?
  ( ) Very Good
  ( ) Fairly Good
  ( ) Fairly Bad
  ( ) Very Bad
6.) During the past month, how often have you taken medicine (**prescribed or "over the counter**") to help you sleep?
  ( ) Not during the past month
  ( ) Less than once a week
  ( ) Once or twice a week
  ( ) Three or more times a week
7.) During the past month, how often have you had trouble staying awake while driving, eating meals, or engaging in social activity?

( ) Not during the past month
( ) Less than once a week
( ) Once or twice a week
( ) Three or more times a week

8.) During the past month, how much of a problem has it been for you to keep up enough enthusiasm to get things done?
( ) No problem at all
( ) Only a very slight problem
( ) Somewhat of a problem
( ) A very big problem

9.) Do you have a bed partner or a roommate?
( ) No bed partner or roommate
( ) Partner/roommate in other room
( ) Partner/roommate in same room, but not same bed
( ) Partner/roommate in same bed

SC: Daily Survey

The following few questions are to help us understand how well you felt that you slept last night, along with your thermal comfort while sleeping with the Pod.
Please respond to the answers as truthfully as possible.

1.) What is your Subject # for this study?

2.) How would you rate the calmness of your sleep last night? [75]
( ) Very Calm (5)
( ) Fairly Calm (4)
( ) Neither calm nor restless (3)
( ) Quite restless (2)
( ) Very restless (1)

3.) How easy was it to fall asleep last night?
( ) Very easy (5)
( ) Fairly easy (4)
( ) Neither easy nor difficult (3)
( ) Quite difficult (2)
( ) Very difficult (1)

4.) How easy was it to wake up this morning?
( ) Very easy (5)
( ) Fairly easy (4)
( ) Neither easy nor difficult (3)
( ) Quite difficult (2)
( ) Very difficult (1)

5.) How refreshed do you feel after waking?
( ) Fully (5)
( ) Fairly (4)
( ) Moderately (3)
( ) Not much (2)

( ) Not at all (1)

6.) How satisfied are you with your sleep last night?
( ) Fully (5)
( ) Fairly (4)
( ) Moderately (3)
( ) Not much (2)
( ) Not at all (1)

7.) On average, throughout the night, what was your thermal sensation? *This is a subjective rating of how cool or hot you felt during the night, on average.*
( ) Cold
( ) Cool
( ) Slightly cool
( ) Neutral
( ) Slightly warm
( ) Warm
( ) Hot

8.) On average, how comfortable were you with your body temperature throughout the night? *This is a rating of your average thermal comfort throughout the night.*
( ) Comfortable
( ) Slightly uncomfortable
( ) Uncomfortable
( ) Very uncomfortable

9.) Did you have any issues with the Sleep Device (HST) device last night? *This would include wires becoming disconnected, trouble with the setup, the battery dying, discomfort, etc.*
( ) Yes
( ) No
( ) NA- I did not wear the HST last night.

10.) Please describe your issue with the Sleep Device (HST)

11.) Did you sleep with the Pod's temperature control on or off last night?
( ) On
( ) Off
( ) NA- did not sleep on the Pod last night.

12.) How do you feel that you slept with Pod temperature control settings compared to your normal mattress? *Please do not account for the Sleep device (i.e., HST) as we know this may have caused some disturbances.*
( ) Slightly better with Pod
( ) Much better with Pod
( ) No difference
( ) Slightly worse with Pod
( ) Much worse with Pod
( ) Cannot tell because of the Home Sleep Test device.

13.) Did you do anything outside of your normal routine last night that may have affected your sleep (other than sleeping on the Pod)? *This may include the consumption of alcohol, having caffeine before bed, a late meal, late exercise, etc.*

( ) Yes

( ) No

14.) Please describe what was outside of your normal routine.

SD: post-Pod PSQI

The following questionnaire has 11 questions to ask about your last week sleeping on the Pod (with the temperature control ON).

These questions relate to your usual sleep habits during the past week. Your answers should indicate the most accurate reply for the majority of the days and nights in the past week. Please answer all questions. Please reach out to our team at rd.clinical@eightsleep.com with any questions.

1.) What is your subject #?
2.) During the past week, when did you usually go to bed at night?
3.) During the past week, how long (IN MINUTES) has it usually taken you to fall asleep each night?
4.) During the past week, when did you usually get up in the morning?
5.) During the past week, how many hours of actual sleep did you get a night? (This may be different than the number of hours you spend in bed.)

INSTRUCTIONS:
For each of the remaining questions, select the best response. Please answer all questions.

6.) During the past week of sleeping on the Pod (with temperature control), how would you rate your sleep quality?

( ) Very Good

( ) Fairly Good

( ) Fairly Bad

( ) Very Bad

7.) During the past week of sleeping on the Pod (with temperature control), how often did you take medicine (prescribed or "over the counter") to help you sleep?

( ) Not during the past week

( ) Once or twice

( ) Three or more times

8.) During the past week of sleeping on the Pod (with temperature control), how often have you had trouble staying awake while driving, eating meals, or engaging in social activity?

( ) Not during the past week

( ) Once or twice

( ) Three or more times

9.) During the past week of sleeping on the Pod (with temperature control), how much of a problem has it been for you to keep up enough enthusiasm to get things done?

( ) No problem at all
( ) Only a very slight problem
( ) Somewhat of a problem
( ) A very big problem

10.) Do you have a bed partner or a roommate?

( ) No bed partner or roommate
( ) Partner/roommate in other room
( ) Partner/roommate in same room, but not same bed
( ) Partner/roommate in same bed
